# Evolution of Fitness Cost-Neutral Mutant PfCRT Conferring *P*. *falciparum* 4-Aminoquinoline Drug Resistance Is Accompanied by Altered Parasite Metabolism and Digestive Vacuole Physiology

**DOI:** 10.1371/journal.ppat.1005976

**Published:** 2016-11-10

**Authors:** Stanislaw J. Gabryszewski, Satish K. Dhingra, Jill M. Combrinck, Ian A. Lewis, Paul S. Callaghan, Matthew R. Hassett, Amila Siriwardana, Philipp P. Henrich, Andrew H. Lee, Nina F. Gnädig, Lise Musset, Manuel Llinás, Timothy J. Egan, Paul D. Roepe, David A. Fidock

**Affiliations:** 1 Department of Microbiology and Immunology, Columbia University Medical Center, New York, New York, United States of America; 2 Division of Pharmacology, Department of Medicine, University of Cape Town, Cape Town, South Africa; 3 Department of Biological Sciences, University of Calgary, Calgary, Alberta, Canada; 4 Departments of Chemistry and of Biochemistry and Cellular & Molecular Biology, Georgetown University, Washington, DC, United States of America; 5 Laboratoire de Parasitologie, WHO Collaborating Center for Surveillance of Anti-Malarial Drug Resistance, Institut Pasteur de la Guyane, Cayenne, French Guiana; 6 Departments of Biochemistry & Molecular Biology and Chemistry, Center for Malaria Research and Center for Infectious Diseases Dynamics, Pennsylvania State University, University Park, Pennsylvania, United States of America; 7 Department of Chemistry, University of Cape Town, Cape Town, South Africa; 8 Division of Infectious Diseases, Department of Medicine, Columbia University Medical Center, New York, New York, United States of America; National Institutes of Health, UNITED STATES

## Abstract

Southeast Asia is an epicenter of multidrug-resistant *Plasmodium falciparum* strains. Selective pressures on the subcontinent have recurrently produced several allelic variants of parasite drug resistance genes, including the *P*. *falciparum* chloroquine resistance transporter (*pfcrt*). Despite significant reductions in the deployment of the 4-aminoquinoline drug chloroquine (CQ), which selected for the mutant *pfcrt* alleles that halted CQ efficacy decades ago, the parasite *pfcrt* locus is continuously evolving. This is highlighted by the presence of a highly mutated allele, Cam734 *pfcrt*, which has acquired the singular ability to confer parasite CQ resistance without an associated fitness cost. Here, we used *pfcrt*-specific zinc-finger nucleases to genetically dissect this allele in the pathogenic setting of asexual blood-stage infection. Comparative analysis of drug resistance and growth profiles of recombinant parasites that express Cam734 or variants thereof, Dd2 (the most common Southeast Asian variant), or wild-type *pfcrt*, revealed previously unknown roles for PfCRT mutations in modulating parasite susceptibility to multiple antimalarial agents. These results were generated in the GC03 strain, used in multiple earlier *pfcrt* studies, and might differ in natural isolates harboring this allele. Results presented herein show that Cam734-mediated CQ resistance is dependent on the rare A144F mutation that has not been observed beyond Southeast Asia, and reveal distinct impacts of this and other Cam734-specific mutations on CQ resistance and parasite growth rates. Biochemical assays revealed a broad impact of mutant PfCRT isoforms on parasite metabolism, including nucleoside triphosphate levels, hemoglobin catabolism and disposition of heme, as well as digestive vacuole volume and pH. Results from our study provide new insights into the complex molecular basis and physiological impact of PfCRT-mediated antimalarial drug resistance, and inform ongoing efforts to characterize novel *pfcrt* alleles that can undermine the efficacy of first-line antimalarial drug regimens.

## Author Summary

Point mutations in the *Plasmodium falciparum* chloroquine resistance transporter (PfCRT) earlier thwarted the clinical efficacy of chloroquine, the former gold standard, and constitute a major determinant of parasite susceptibility to antimalarial drugs. Recently, we reported that the highly mutated Cambodian PfCRT isoform Cam734 is fitness-neutral in terms of parasite growth, unlike other less fit isoforms such as Dd2 that are outcompeted by wild-type parasites in the absence of CQ pressure. Using *pfcrt*-specific zinc-finger nucleases to genetically dissect the Cam734 allele, we report that its unique constituent mutations directly contribute to CQ resistance and collectively offset fitness costs associated with intermediate mutational steps. We also report that these mutations can contribute to resistance or increased sensitivity to multiple first-line partner drugs. Using isogenic parasite lines, we provide evidence of changes in parasite metabolism associated with the Cam734 allele compared to Dd2. We also observe a close correlation between CQ inhibition of hemozoin formation and parasite growth, and provide evidence that Cam734 PfCRT can modulate drug potency depending on its membrane electrochemical gradient. Our data highlight the capacity of PfCRT to evolve new states of antimalarial drug resistance and to offset associated fitness costs through its impact on parasite physiology and hemoglobin catabolism.

## Introduction

Human malaria remains a leading global health scourge in part due to multidrug resistance mechanisms evolved by *Plasmodium falciparum*, the protozoan species responsible for the most severe forms of disease [1]. Artemisinin-based combination therapies (ACTs) are the current first-line means of controlling pathogenic asexual blood-stage infections, including ones dominated with drug-resistant strains that arose during previous selective sweeps resulting from the global use of chloroquine (CQ) and sulfadoxine-pyrimethamine [2–4]. The 4-aminoquinoline compound CQ was especially pivotal earlier in reducing mortality rates [5]. However, the multi-focal emergence and spread of CQ resistance (CQR) contributed to stalled control measures and substantial increases in malaria-associated hospitalizations and deaths [6]. Nevertheless, owing to its safety, affordability, and established efficacy against non-resistant parasites, CQ continues to be deployed in regions that are free of CQR or that harbor CQ-sensitive *P*. *vivax* [7]. Interestingly, studies of infections with CQ-resistant *P*. *falciparum* strains in Guinea-Bissau recently revealed a ~5–fold increase in CQ efficacy upon doubling the standard dose in children aged <5 years, the age demographic at highest risk for malaria mortality [8]. These findings coincide with renewed efforts to delineate the molecular basis of resistance to antimalarials bearing the hallmark CQ-type quinoline moiety [9].

Genetic linkage and allelic replacement studies have previously identified *pfcrt* variants as the primary determinant of CQR [10,11]. These findings are supported by evidence of directional selection for mutant *pfcrt* alleles in *P*. *falciparum* parasite populations subjected to extensive CQ pressure [12]. A secondary, strain-dependent contribution to CQR has also been noted for the *P*. *falciparum* multidrug resistance 1 (*pfmdr1*) gene [13–15]. Among CQ-resistant field isolates, PfCRT isoforms are comprised of geographically distinct clusters of single-nucleotide polymorphisms (SNPs), namely K76T and 3 to 8 additional point mutations. PfCRT K76T is a critical, albeit insufficient, determinant of parasite *in vitro* CQR [16]. This mutation also predicts *in vivo* CQ treatment failure with high sensitivity but lower specificity [17]. At the cellular level, PfCRT is a multi-pass transporter embedded in the intra-erythrocytic parasite’s digestive vacuole (DV) membrane, with enigmatic functions that may include transport of ions and/or peptides [18–21]. In the absence of PfCRT structural information, mutational approaches have guided studies into the effect of specific PfCRT mutations on drug transport and parasite growth [16,22–24].

Point mutations in PfCRT have also been associated with altered parasite susceptibility to ACT component drugs, namely artemisinins and their partner drugs (including amodiaquine, lumefantrine, and piperaquine) [25–29]. This is of particular relevance given reports of emerging clinical resistance to these first-line agents [30,31]. To various degrees, these compounds interfere with or are otherwise impacted by parasite-mediated catabolism of host hemoglobin (Hb), which supplies parasites with amino acids and helps maintain intracellular osmolarity [32–35]. This catabolic process produces ferriprotoporphyrin IX heme, which in its reactive free form can exert lethal oxidative damage to the parasite [36]. For quinoline-based antimalarials, drug-heme interactions in the DV cause toxicity by preventing incorporation of ferriprotoporphyrin IX heme dimers (β-hematin) into the non-reactive hemozoin (Hz) crystals that account for >95% of total heme [37,38]. Consistent with this inhibition of β-hematin mineralization and detoxification, CQ treatment of drug-sensitive D10 parasites was recently observed by transmission electron microscopy to disrupt the highly ordered fringe pattern of Hz crystals [32].

Cell fractionation methods in *P*. *falciparum* D10 parasites have further demonstrated that, upon CQ treatment, the proportion of total heme present as Hz significantly diminishes, whereas the proportion corresponding to free heme increases [32]. These responses are dose-dependent and inversely proportional to parasite survival [32]. As a weak base and lipophilic drug, CQ traverses multiple lipid bilayers and accumulates as CQ^2H+^ up to a thousand-fold in the acidic DV, where it binds hematin, hemozoin, or both [39]. CQR-promoting PfCRT isoforms appear to efficiently transport CQ out of the DV, consequently restricting CQ-heme contacts and allowing Hz formation to proceed [7,21]. Of note, the mutational status of *pfcrt* can also impact DV volume and pH, both of which influence Hz formation kinetics [40].

Interestingly, recent metabolomic analyses of CQ-resistant versus CQ-sensitive *P*. *falciparum* strains detected a link between mutant *pfcrt*-mediated CQR and the elevated accumulation of peptides derived from Hb digestion [41]. Given the reduced growth of CQ-resistant parasites (expressing the Dd2 or 7G8 mutant *pfcrt* alleles) relative to recombinant isogenic parasites encoding wild-type *pfcrt*, defective Hb degradation was postulated as a cellular basis for the reduced fitness associated with mutant *pfcrt* [41]. Reduced fitness of these mutant alleles was confirmed in *in vitro* cell culture studies [41,42] and was observed at a population level in Africa, where the removal of CQ pressure led to the attrition of mutant *pfcrt*-expressing parasites in favor of wild-type, CQ-sensitive strains [43,44].

A pathogen’s fitness refers to its capacity to support infection and generate new progeny. For *P*. *falciparum* parasites, fitness is influenced in part by the rate of growth of pathogenic asexual blood-stage parasites, selective forces exerted by drug pressure, mosquito-human transmission, and selection within the mosquito vector [41,44,45]. In general, these factors are impaired in parasites expressing mutant, CQR-associated PfCRT isoforms [7,46]. To date, over 50 distinct PfCRT haplotypes have been reported [22]. Of these, the Asian haplotype Dd2 (M74I/N75E/K76T/A220S/Q271E/N326S/I356T/R371I) and the South American/Western Pacific haplotype 7G8 (C72S/K76T/A220S/N326D/I356L) account for a large proportion of global mutant types, with additional isoforms harboring four or more SNPs and resembling the PfCRT haplotypes Dd2 or 7G8 (notably the six-SNP African variant GB4 or the four-SNP South American variant Ecu1110 respectively) [7]. Recent modeling in *P*. *falciparum* suggests that *pfcrt* evolution occurred via punctuated periods of mutation that were too brief to allow fixation of partially mutated alleles (i.e. bearing 1 to 3 SNPs), shedding light on physiologic constraints that explain the rarity of mutant *pfcrt* emergence in the field [16]. Intriguingly, studies from Cambodia, an epicenter of multidrug resistance in *P*. *falciparum*, also revealed a highly polymorphic CQR-conferring *pfcrt* allele, Cam734 [47]. This allele encodes nine mutations (see Table 1), five of which (N75D, A144F, L148I, I194T, T333S) are not found in the predominant Southeast Asian CQ-resistant *pfcrt* allele, Dd2 [22]. After Dd2, Cam734 *pfcrt* represents the second most prevalent allele in Southeast Asia [27]. Unlike other CQR-associated isoforms such as Dd2, the Cam734 allele has been found to be fitness-neutral in that it supports parasite growth comparable to recombinant *pfcrt*-edited parasites (engineered on the same strain, and referred to as isogenic) that encode the CQ-sensitive, wild-type *pfcrt* allele [42].

**Table 1:** PfCRT status of lines employed in this study.

			PfCRT residue											
Parasite Line	PfCRT haplotype	Rec.	74	75	76	144	148	194	220	271	326	333	356	371
GC03^Cam734^	Cam734	Yes	I	D	T	F	I	T	S	E	N	S	I	R
GC03^Cam734 D75N^	Cam734 D75N	Yes	I	N	T	F	I	T	S	E	N	S	I	R
GC03^Cam734 F144A^	Cam734 F144A	Yes	I	D	T	A	I	T	S	E	N	S	I	R
GC03^Cam734 I148L^	Cam734 I148L	Yes	I	D	T	F	L	T	S	E	N	S	I	R
GC03^Cam734 T194I^	Cam734 T194I	Yes	I	D	T	F	I	I	S	E	N	S	I	R
GC03^Cam734 S333T^	Cam734 S333T	Yes	I	D	T	F	I	T	S	E	N	T	I	R
GC03^Dd2^	Dd2	Yes	I	E	T	A	L	I	S	E	S	T	T	I
Dd2	Dd2	No	I	E	T	A	L	I	S	E	S	T	T	I
GC03^GC03^	GC03 (wild-type)	Yes	M	N	K	A	L	I	A	Q	N	T	I	R
GC03	GC03 (wild-type)	No	M	N	K	A	L	I	A	Q	N	T	I	R

Dark gray shading denotes mutations specific to Cam734. Light gray shading denotes mutations present in Dd2, some of which are shared with Cam734. PfCRT haplotypes Cam734 F144A and Cam738 are equivalent. Rec., recombinant.

This unique Cam734 PfCRT isoform presents an opportunity to explore *P*. *falciparum* genetic determinants that concurrently confer drug resistance and fully neutralize fitness costs, a unique feature not associated with other mutant PfCRT variants. Herein, we leveraged drug resistance versus growth profiling of isogenic, *pfcrt*-modified asexual blood-stage parasites. These studies were combined with biochemical approaches—including metabolomic, heme fractionation, and heterologous expression studies—in order to address the following questions: (1) To what extent do the mutations unique to Cam734 PfCRT directly impact parasite resistance to clinically employed antimalarials? (2) Which mutations are compensatory and thus serve to preserve PfCRT function? and (3) Mechanistically, how does mutant Cam734 PfCRT confer CQR without an accompanying fitness cost? Results provided herein broaden our present understanding of the mechanistic basis of CQR and inform field efforts that evaluate *pfcrt* genotypes as a tool to predict the drug susceptibility status of clinical isolates.

## Results

### Generation of isogenic parasites encoding full-length or back-mutated Cam734 *pfcrt* alleles

To dissect the contributions of the rare mutations comprising Cam734 PfCRT to parasite drug resistance and fitness, we utilized a recently established [48] gene-editing approach (S1 Fig) with *pfcrt*-specific zinc-finger nucleases (ZFNs). Starting with the GC03 strain, a CQ-sensitive progeny of the HB3×Dd2 genetic cross [49], we engineered isogenic parasites encoding full-length Cam734 *pfcrt* (GC03^Cam734^; the PfCRT haplotype of recombinant lines is listed in superscript) as well as partial Cam734-like isoforms containing “back-to-wild-type” mutations at PfCRT residues 75, 144, 148, 194, and 333 (see Table 1). Our parasite panel also included the GC03^Dd2^ and GC03^GC03^ lines, which encode the mutant Dd2 (CQ-resistant) and wild-type GC03 (CQ-sensitive) haplotypes, respectively and which were similarly engineered using ZFN-based editing (see Table 1). This ZFN approach enables the expression of only full-length *pfcrt*, and is thus a significant improvement over the prior allelic exchange method [11] that in addition to expressing full-length *pfcrt* also generated truncated fragments that created the possibility of internal recombination events. We note that the host GC03 strain has been used in multiple prior *pfcrt* allelic exchange and gene editing studies [11,16,42,48,50], providing extensive background data on parasite drug susceptibilities, transport properties, transcriptional changes, drug-heme interactions and metabolomics [41,51–59]. For each recombinant line, two independent clones were selected, and *pfcrt* sequence integrity was verified using sequencing primers listed in S1 Table. Recombinant parasite design and validation, including PCR-based confirmation of the recombinant locus, cDNA sequencing that showed error-free editing, and Western blot analysis confirming equivalent expression, is further detailed in S1 Fig and **Supplementary Materials and Methods**.

### Cam734 *pfcrt-*defining mutations impact parasite responses to chloroquine

We examined the roles of Cam734 PfCRT-constituent mutations in mediating CQR by assessing the responses of recombinant, *pfcrt*-modified parasites to CQ and its clinically relevant metabolite, monodesethyl-CQ (md-CQ). Md-CQ was included in our analysis as it shows a greater distinction between CQ-sensitive and CQ-resistant lines and may have been the primary evolutionary selective agent [11,16]. For all drug assays, genetically unmodified Dd2 and GC03 parasites were included as reference lines (see Table 1). Using 72 h flow cytometry-based drug susceptibility assays, we determined antimalarial drug concentrations that result in 50% (IC_50_) and 90% (IC_90_) inhibition of parasite proliferation (S2 Table). Both values have earlier proven informative in defining CQ susceptibility phenotypes, particularly in cases of low-level resistance or tolerance [50]. Statistical comparisons were performed against GC03^Cam734^ parasites, which express the full-length Cam734 *pfcrt* allele. In our analysis, recombinant GC03 parasites encoding the major Southeast Asian *pfcrt* variants Cam734 and Dd2 conferred moderate (~5-fold and ~14-fold) and high-level (~26-fold and ~43-fold) increases in CQ and md-CQ IC_50_ values, respectively, when compared to CQ-sensitive GC03^GC03^ parasites (S2 Table). This is consistent with earlier *P*. *falciparum* drug susceptibility studies [42].

**Fig 1:**
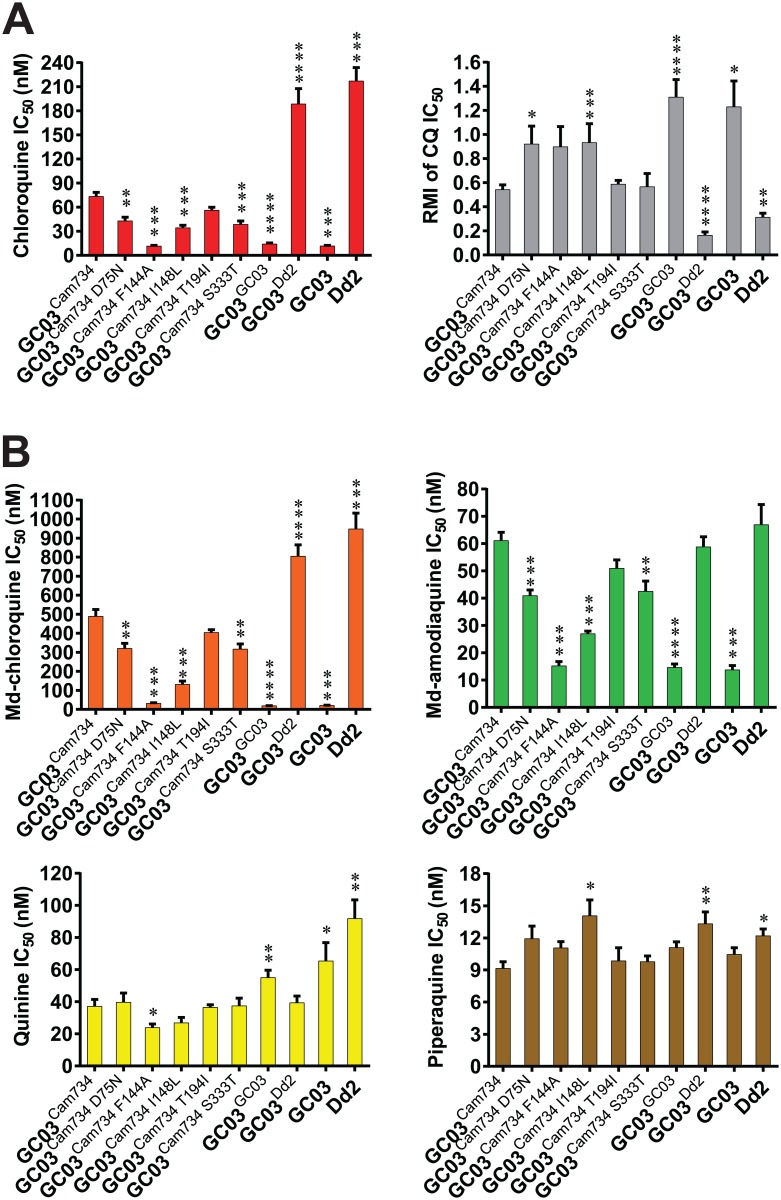
Drug resistance profiles of *pfcrt*-modified and reference parasite lines. **(A)** Parasite chloroquine (CQ) responses and verapamil (VP) reversibility of CQ resistance. Briefly, flow cytometry was used to assess parasitemias and quantify the corresponding drug concentration-dependent inhibition following 72 h exposure to the indicated antimalarial drugs. Bar graphs correspond to mean ± SEM IC_50_ or response modification index (RMI) values. IC_50_ values correspond to the drug concentrations that produced 50% inhibition of parasite growth. The RMI of CQ IC_50_ is equivalent to (IC_50_ for CQ + VP) ÷ (IC_50_ for CQ only), as detailed in **Materials and Methods**. **(B)** Parasite responses to monodesethyl (md)-chloroquine, md-amodiaquine, quinine, and piperaquine. Bar graphs indicate mean ± SEM IC_50_ values. Results encompass 3 to 12 independent assays conducted in duplicate. Statistical differences were determined via non-parametric Mann-Whitney *U* tests, using the mean IC_50_ value of Cam734 *pfcrt*-expressing GC03^Cam734^ parasites as the comparator. CQ susceptibility and CQ RMI data are summarized along with corresponding statistical tests in S2 and S3 Tables, respectively. IC_90_ values and Hill slopes are also presented in S2 Table. *P<0.05; **P<0.01; ***P<0.001. ****P<0.0001.

CQ (Fig 1A; S2 Table) and md-CQ (Fig 1B; S2 Table) susceptibility profiles revealed significant roles for multiple Cam734 PfCRT-defining mutations in conferring CQR. Among these, PfCRT A144F was indispensable for CQ and md-CQ resistance (compare GC03^Cam734^ with the GC03^Cam734 F144A^ line). Indeed, removal of this mutation restored complete to near-complete sensitivity to CQ and md-CQ respectively, at IC_50_ and IC_90_ levels (S2 Table). The Cam734 F144A PfCRT haplotype (see Table 1) is equivalent to Cam738 PfCRT, which, like Cam734, was initially documented in Cambodia but, in contrast, did not achieve wide regional spread [47]. Notably, although the Cam734 F144A haplotype harbors K76T and seven additional mutations, its mutational configuration is nevertheless insufficient for CQR (see Fig 1A and S2 Table). This underscores the notion of K76T as an insufficient predictor of CQR status and supports the fact that CQ resistance or susceptibility can depend on additional PfCRT substitutions (e.g. C101F, L272F, C350R, or in this case A144F), even when K76T is present [22,60–62].

Our analysis identified additional contributory roles for PfCRT mutations N75D, L148I, and T333S in conferring parasite resistance to CQ (Fig 1A; S2 Table) and md-CQ (Fig 1B; S2 Table), as parasite lines encoding back-mutations at each of the corresponding PfCRT residues demonstrated significant reductions in resistance (range of 1.5 to 3.7-fold reductions in CQ or md-CQ IC_50_ values for GC03^Cam734 D75N^, GC03^Cam734 I148L^, and GC03^Cam734 S333T^ as compared to GC03^Cam734^ parasites). Thus, to various degrees, N75D, A144F, L148I, and T333S directly contribute to CQR and are not merely compensatory in nature in terms of restoring function or fitness to mutant PfCRT isoforms. Only the I194T mutation was found to not significantly contribute to CQR.

A defining molecular feature of *P*. *falciparum* CQR is resistance reversal by the calcium channel blocker verapamil (VP) [52]. The extent to which VP modifies CQR, referred to as the CQ response modification index (RMI), is PfCRT isoform-specific and is calculated by dividing the IC_50_ value for CQ in the presence of 0.8 μM VP by the IC_50_ value for CQ alone [63]. Our CQR reversibility results are depicted in Fig 1A and S3 Table. Consistent with previous studies [42], isogenic parasites expressing PfCRT variants Cam734 (GC03^Cam734^) and Dd2 (GC03^Dd2^) exhibited moderate and high-level CQR reversibility (2.4-fold and 8.1-fold reductions in the CQ RMI, respectively, versus the GC03^GC03^ line that showed no CQR reversibility). Among the recombinant lines encoding partial, back-to-wild-type Cam734 PfCRT haplotypes, GC03^Cam734 D75N^ and GC03^Cam734 I148L^ parasites exhibited statistically significant increases in CQ RMI values as compared to GC03^Cam734^ parasites, highlighting critical roles for mutations N75D and L148I in the VP reversibility effect. These findings uncover a novel role for PfCRT residue 148 in mediating VP reversal of CQR and align with previous studies that implicate an important role for mutations at residue 75 in mediating this reversal phenotype [42,64].

### Cam734 *pfcrt-*defining mutations impact parasite responses to clinically important antimalarials

PfCRT variants can modulate parasite susceptibility to a host of antimalarials beyond CQ [7]. We consequently assessed the effects of Cam734 PfCRT-constituent mutations on parasite responses to various clinically employed antimalarials (Fig 1 and S2 Fig). Our drug panel consisted of the following: (1) ACT partner drugs, including monodesethyl-amodiaquine (md-AQ, the active metabolite of AQ), lumefantrine (LUM), piperaquine (PPQ), and pyronaridine (PND); (2) the ACT artemisinin derivative artesunate (AS); and (3) quinine (QN), a second-line agent used to treat severe malaria. In keeping with known cross-resistance relationships between CQ and AQ [7], we observed significant reductions in md-AQ resistance (Fig 1B; S2 Table) among parasites harboring reversions of Cam734 PfCRT mutations N75D, A144F, L148I, and T333S (range of 1.4 to 4.0-fold reductions in md-AQ IC_50_ values for GC03^Cam734 D75N^, GC03^Cam734 F144A^, GC03^Cam734 I148L^, and GC03^Cam734 S333T^ as compared to GC03^Cam734^ parasites). Reversion of the PfCRT mutation A144F back to wild-type (compare GC03^Cam734^ with GC03^Cam734 F144A^) was likewise associated with a significant (~1.6-fold) reduction in QN IC_50_ values (Fig 1B; S2 Table), emphasizing A144F as a critical determinant of parasite resistance to multiple quinoline-type antimalarials. We further detected a modest (~1.5-fold), although statistically significant, increase in PPQ IC_50_ for GC03^Cam734 I148L^ parasites, as compared to GC03^Cam734^ parasites. This highlights the capacity of PfCRT mutations to impact parasite PPQ resistance, a rising problem in Southeast Asia with a presently unclear genetic basis [30,65,66]. As compared to wild-type (GC03) *pfcrt*, full-length Cam734 sensitized parasites to the antimalarial compounds LUM, AS, and PND, and this phenotype was not modulated by any of the Cam734-constituent mutations studied herein (S2 Fig; S2 Table).

### Cam734 *pfcrt-*defining mutations offset parasite fitness costs *in vitro*

**Fig 2:**
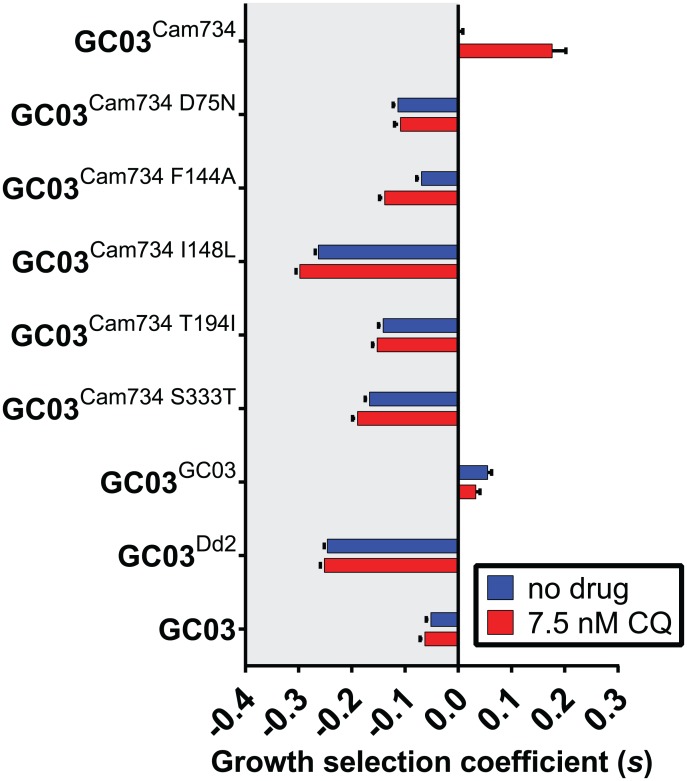
*In vitro* growth profiles of *pfcrt*-modified and reference parasite lines. Briefly, co-cultures initially consisting of a 1:1 ratio of a GFP^−^ test line and a GFP^+^ reporter line were monitored by flow cytometry each 48 h generation for 10 generations (see Materials and Methods and S3 Fig), and the per-generation selection coefficient (*s*) for each test line was derived from parasite growth curves (see **Supplementary Materials and Methods**). Bar graphs correspond to mean ± SEM *s* values for parasites subjected to no drug or 7.5 nM chloroquine (CQ). A summary of *s* values and inter- and intra-strain statistical tests is provided in S4 Table.

Previous efforts to disrupt the *pfcrt* gene demonstrated that it is essential for survival of asexual blood-stage *P*. *falciparum* parasites [67]. Furthermore, PfCRT mutations can be deleterious to parasite growth [16,42], a measurable phenotype that serves as a proxy for fitness and reflects parasite functional requirements [68]. To evaluate the contributions of Cam734-unique PfCRT mutations to parasite growth, we used previously established co-culture methods to derive relative growth estimates [16,69]. Co-cultures consisted of equal proportions of a *pfcrt*-modified GFP-negative (GFP^−^) test line and a wild-type *pfcrt*-expressing GFP-positive (GFP^+^) reporter line (see Materials and Methods). To assess the impact of a sub-therapeutic dose of CQ on parasite growth, experiments were performed in the absence or presence of 7.5 nM CQ (~0.5× CQ IC_50_ of CQ-sensitive GC03^GC03^ parasites; see S2 Table). These co-cultures were monitored for 10 generations, with the GFP^−^ proportion of the co-culture determined every 48 h generation by flow cytometry (S3 Fig). As detailed in **Materials and Methods** and **Supplementary Materials and Methods**, these data were used to derive the per-generation selection coefficient (*s*) of each test line (Fig 2; S4 Table). This coefficient serves as a proxy for the degree of fitness of a given parasite line, as compared to GC03^Cam734^ parasites, which encode the full-length Cam734 *pfcrt* allele (*s* = 0, *s*>0, and *s*<0 indicate fitness levels equal, greater than, or less than that of GC03^Cam734^ parasites).

As determined in our *in vitro* growth assays (Fig 2; S4 Table), the growth rate of parasites encoding Cam734 *pfcrt* was markedly increased relative to isogenic parasites encoding mutant Dd2 *pfcrt* (*s* = -0.25 for GC03^Dd2^ versus GC03^Cam734^) and was more comparable to growth of parasites encoding wild-type GC03 *pfcrt* (*s* = 0.06 for GC03^GC03^ versus GC03^Cam734^). These findings agree with earlier studies of isogenic, *pfcrt*-modified lines constructed via an independent allelic exchange strategy [42]. Remarkably, growth of GC03^Cam734^ parasites was significantly enhanced (~1.2–fold; Fig 2; S4 Table) by the presence of a sub-lethal dose of CQ (7.5 nM), surpassing the growth rates of all other parasite lines, including those expressing GC03 *pfcrt*. Our results further demonstrate that the Cam734 PfCRT-constituent mutations N75D, A144F, L148I, I194T, and T333S distinctly contribute to the growth of asexual blood-stage parasites expressing full-length Cam734 PfCRT, as the corresponding partial, back-mutant haplotypes conferred significantly decreased relative growth rates (with *s* values ranging from -0.07 to -0.30 for GC03^Cam734 D75N^, GC03^Cam734 F144A^, GC03^Cam734 I148L^, GC03^Cam734 T194I^, and GC03^Cam734 S333T^; see S4 Table).

Among the back-mutant parasites, the most deleterious growth was observed for parasites expressing Cam734 I148L PfCRT, which was reminiscent of the growth phenotype of the Dd2 PfCRT isoform that is known to substantially impair parasite fitness (compare GC03^Cam734 I148L^ and GC03^Dd2^; Fig 2; S4 Table). Considering our dissection of the roles of Cam734 PfCRT mutations in parasite CQ susceptibility and growth, our results suggest that PfCRT mutations N75D, A144F, I148L, and S333T play dual roles, directly contributing to CQR and compensating for associated fitness costs, whereas I194T has no impact on CQR and only helps improve growth.

### Cam734 *pfcrt* affects hemoglobin processing and central carbon metabolism

The catabolism of host-derived Hb is an essential parasite process that liberates two major products: (1) free heme that is subsequently incorporated into crystalline Hz; and (2) free Hb-derived peptides that can contribute to the parasite’s nutrient pool [70]. Recent studies have shown that peptides derived from either the α or β chains of Hb can accumulate up to 32–fold within isogenic parasites expressing CQ-resistant Dd2 or 7G8 *pfcrt* alleles as compared to CQ-sensitive (wild-type) *pfcrt*-expressing parasites [41]. The accumulation of Hb-derived peptides in parasites expressing PfCRT Dd2 or 7G8 variants was linked to impaired Hb catabolism and was proposed to be a causal determinant of their reduced fitness, as measured using *in vitro* growth rates [41]. Given the unique capacity of mutant Cam734 PfCRT to neutralize fitness costs that are typically associated with CQ-resistant PfCRT isoforms, we examined whether Cam734 PfCRT mitigated the accumulation of Hb-derived peptides.

**Fig 3:**
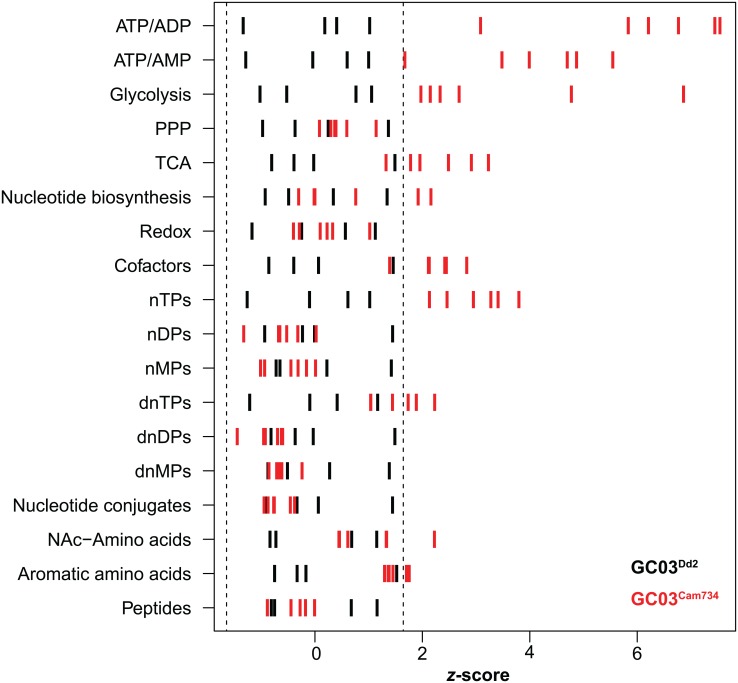
Metabolomic profiles of isogenic, mutant *pfcrt*-expressing parasites. Metabolite extracts derived from tightly synchronized trophozoite-stage isogenic (GC03) parasites encoding either Dd2 or Cam734 *pfcrt* were analyzed by mass spectrometry. For each metabolite class, individual metabolite signals were expressed as *z*-scores (detailed in Materials and Methods), allowing for direct comparisons across distinct metabolite classes. Dashed lines represent lower (5%) and upper (95%) boundaries for the normal distribution, as defined for GC03^Dd2^ (black) parasites. Metabolites were harvested on three independent occasions (*n* = 4 to 6 total replicates per parasite strain). Compound class abbreviations, *z*-scores, and *P* values are presented in S6 Table.

To test this, we measured endogenous metabolite levels in isogenic lines encoding the CQ-resistant *pfcrt* alleles Cam734 and Dd2 (GC03^Cam734^ and GC03^Dd2^). Briefly, red blood cells (RBCs) harboring late-stage (~36–42 h) trophozoites were magnetically purified, metabolites were extracted, and extracts were analyzed using established mass spectrometry-based metabolomic methods [41]. Results are depicted in Fig 3 for compound classes and S4 Fig for individual metabolites. Metabolite signal intensities and *z*-scores are reported in S5 and S6 Tables, respectively. Our results show that the CQ-resistant *pfcrt* alleles Cam734 and Dd2 accumulated comparable levels of Hb-derived peptides (*P* = 0.33; see Fig 3 and S6 Table). Moreover, the GC03^Cam734^ and GC03^Dd2^ peptide levels were significantly elevated relative to genetically matched GC03^GC03^ parasites expressing the wild-type *pfcrt* [41]. Consequently, peptide accumulation in these lines was not correlated with the observed differences in fitness between Cam734 and Dd2.

To better understand the impact of the Cam734 allele, we conducted a more comprehensive metabolic analysis of GC03^Cam734^ and GC03^Dd2^ parasites. This analysis revealed several distinguishing metabolic phenotypes. Most significantly, ATP to ADP and ATP to AMP ratios were significantly higher in GC03^Cam734^ compared to GC03^Dd2^ parasites (*P* = 0.0002 and *P* = 0.0001, respectively; see Fig 3 and S6 Table). Low ATP to AMP ratios are a classic marker of lower cellular energy and metabolic stress [71]; the relatively higher ratios seen in GC03^Cam734^ parasites are consistent with their increased *in vitro* growth rate as compared with GC03^Dd2^ (see Fig 2). GC03^Cam734^ parasites also exhibited significantly increased levels of glycolytic and tricarboxylic acid (TCA) cycle-associated metabolites (*P* = 0.003 and *P* = 0.01, respectively; see Fig 3 and S6 Table). These elevated central carbon metabolites may suggest that GC03^Cam734^ achieves its altered energy state via a metabolic compensatory mechanism. Collectively, these findings indicate that parasites encoding Cam734 and Dd2 PfCRT both suffer from impaired parasite Hb catabolism, but that the Cam734 PfCRT isoform compensates for this defect via a mechanism that may involve alterations in central carbon metabolism.

### Distribution of heme species in isogenic *pfcrt*-modified parasites

**Fig 4:**
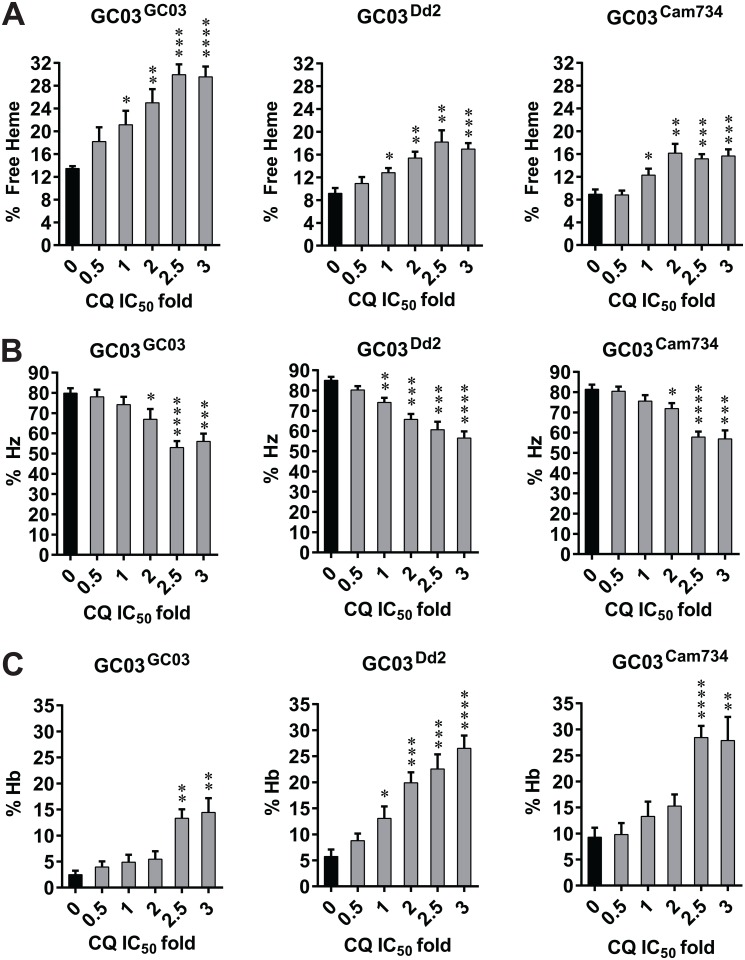
Distribution of heme species in control and chloroquine (CQ)-treated *pfcrt*-modified parasite lines. The percent of total heme present as **(A)** free heme, **(B)** hemozoin (Hz), or **(C)** hemoglobin (Hb) was measured spectrophotometrically in recombinant isogenic GC03 parasites expressing the wild-type (CQ-sensitive) GC03 *pfcrt* allele or mutant (CQ-resistant) Dd2 or Cam734 *pfcrt* alleles. Prior to heme fractionation, synchronous parasites were exposed for 32 h to multiples of strain-specific CQ IC_50_ values (1× IC_50_ values for GC03^GC03^, GC03^Dd2^, and GC03^Cam734^ in these experiments were 19.5 nM, 187 nM, and 90.9 nM, respectively). Bar graphs indicate mean ± SEM percentage values for 5 to 10 independent replicates. For each parasite line, values obtained for CQ-treated samples (gray bars) were compared against the untreated control (black bars), and statistical significance was determined via unpaired *t* tests with Welch’s correction. Absolute amounts of heme species as a function of CQ concentrations are depicted in S5 Fig. *P<0.05; **P<0.01; ***P<0.001. ****P<0.0001.

CQ treatment of *P*. *falciparum* parasites affects their disposition of the different forms (“species”) of heme, namely free heme, Hz, and Hb. In a dose-dependent manner, CQ causes an increase in toxic free heme, a decrease in the formation of chemically inert Hz crystals, and an accompanying reduction in parasite survival [32]. To date, the profiles of heme fractions have only been explored in CQ-sensitive (D10, NF54 and D6) parasites [72,73]. Given the central role of *pfcrt* in dictating parasite responses to CQ, we examined the composition of heme species in CQ-treated and untreated isogenic parasites encoding either the CQ-sensitive (wild-type) *pfcrt* allele GC03 or the CQ-resistant (mutant) *pfcrt* alleles Cam734 or Dd2. Our heme fractionation assay entailed treating synchronized early ring-stage parasites with CQ across a range (0 to 3×) of its IC_50_ values for the different lines. After 32 h, trophozoite-stage parasites were subjected to a series of cellular fractionation steps. The abundance of free heme, Hz and Hb was subsequently determined spectrophotometrically and reported both as a percentage of total heme (Fig 4) and as an amount of heme iron (Fe) per cell (S5 Fig).

Our results demonstrate that parasite exposure to CQ caused a dose-dependent increase in free heme for all *pfcrt*-modified parasite lines. Considering all CQ treatments, the highest accumulation of free heme was observed in the CQ-sensitive GC03^GC03^ parasites, with maximal mean free heme Fe concentrations of 19.3, 12.6, and 10.5 femtograms (fg) per cell observed in GC03^GC03^, GC03^Dd2^, and GC03^Cam734^ parasites respectively (Fig 4A and S5A Fig). The difference between GC03^GC03^ and GC03^Cam734^ parasites achieved statistical significance (*P* = 0.02 by unpaired *t* test with Welch’s correction). Inversely correlating with free heme profiles, Hz amounts showed comparable, CQ dose-dependent decreases (Fig 4B and S5B Fig).

We note that GC03^GC03^ parasites showed a higher, statistically significant level of free heme at baseline (i.e. in the absence of CQ) as compared to GC03^Dd2^ and GC03^Cam734^ parasites (*P* = 0.004 and *P* = 0.0012, respectively, by unpaired *t* tests with Welch’s correction), although the lowest level of Hz achieved in all three CQ-treated strains was comparable (Fig 4B). Maximal free heme amounts in the presence of CQ even at very high concentrations were also markedly lower in GC03^Dd2^ and GC03^Cam734^ parasites compared to the CQ-sensitive GC03^GC03^ parasites (S5 Fig).

Several notable differences were also observed among the Hb profiles of *pfcrt*-modified lines (Fig 4C and S5C Fig). First, GC03 parasites encoding wild-type (GC03) *pfcrt* exhibited lower concentrations of Hb at baseline as compared to isogenic parasites expressing the mutant *pfcrt* alleles Dd2 or Cam734 (mean amounts of 2.3, 3.7 and 6.3 Hb fg per cell respectively in untreated samples; S5C Fig), with the difference between the GC03^GC03^ and GC03^Cam734^ lines achieving statistical significance (*P* = 0.006 by unpaired *t* test with Welch’s correction). Consistent with previous findings [32], CQ-sensitive GC03^GC03^ parasites (Fig 4C and S5C Fig) showed a significant elevation in Hb species that did not occur until 2.5× CQ IC_50_. A comparable accumulation in Hb starting at 2.5× CQ IC_50_ was observed for GC03^Cam734^ parasites, contrasting with the profile of GC03^Dd2^ parasites, which showed elevations in Hb amounts at a lower (1×) CQ IC_50_ fold (Fig 4C and S5C Fig). The increase in Hb observed for GC03^Dd2^ parasites coincided with a statistically significant increase in free heme at 1× CQ IC_50_ (see Fig 4A). This is consistent with previous studies of CQ-treated parasites, in which increases in undigested Hb were found to follow significant increases in free heme [32].

**Fig 5:**
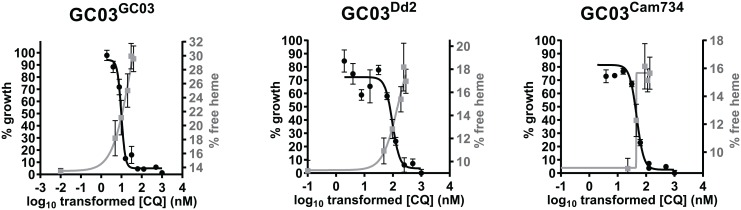
Parasite growth and percentage of free heme as a function of CQ concentration for recombinant isogenic *pfcrt*-modified parasites. Curves show parasite growth (black curve) and percentage of free heme species (gray curve), graphed as a function of the log_10_-transformed CQ concentration (in nM). Plotted points and error bars correspond to mean ± SEM measurements made in parasite growth assays (*n* = 8–10) or heme fractionation assays (*n* = 5–10), as detailed in **Materials and Methods**.

For each *pfcrt*-modified line, we also compared the CQ dose dependence of free heme fractions versus parasite growth (Fig 5). Interestingly, for each line, the free heme concentration curve crossed the parasite growth curve at approximately the same mid-point (IC_50_ value), indicating that the inverse relationship between CQ action on free heme levels and growth inhibition that was previously observed for parasite lines encoding wild-type *pfcrt* [73] is preserved among lines encoding CQ-resistant *pfcrt* alleles. These data provide compelling evidence that for both resistant and sensitive parasites, CQ-mediated growth inhibition results primarily from this drug’s inhibition of Hz formation, which the parasite uses to detoxify reactive free heme.

### Cam734 PfCRT confers membrane potential-sensitive chloroquine transport

A key feature of CQR in *P*. *falciparum* parasites is the ability of mutant PfCRT to efflux CQ from the parasite DV, in turn reducing CQ access to heme. Measurement of drug transport is experimentally challenging in *Plasmodium* parasites due to the presence of multiple membrane-bound intracellular compartments. Accordingly, we evaluated CQ transport mediated by Cam734 PfCRT using a recently optimized *Saccharomyces cerevisiae* galactose-inducible PfCRT expression system [22]. Yeast-expressed PfCRT isoforms localize largely to the cell membrane and mediate transport of CQ from the external medium (low pH, positive membrane potential [Ψ]) to the yeast cytosol (high pH, negative Ψ), recapitulating the electrochemical gradient-driven transport of CQ from the parasite DV (low pH, positive Ψ) to the parasite cytosol (high pH, negative Ψ) [74]. Of note, at baseline, the yeast cell membrane possesses a high ΔpH and a low Δψ. By increasing the pH of the external medium (pH_external_), the cell membrane ΔpH can be experimentally lowered, yielding a compensatory potassium channel-dependent increase in Δψ [74]. In this system, at higher Δψ, CQ transport by CQ-resistant PfCRT isoforms is more pronounced as compared to the basal level of transport mediated by the CQ-sensitive wild-type GC03 PfCRT isoform [22,75]. Earlier studies have validated that growth rates of PfCRT-expressing yeast serve as a useful proxy for CQ transport [75].

**Fig 6:**
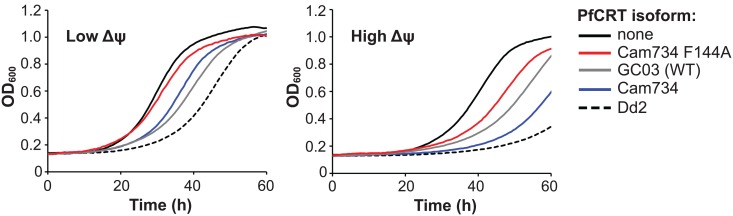
Effect of Δψ on CQ-induced growth inhibition of yeast expressing PfCRT isoforms. Growth (measured as OD_600_) of yeast harboring no (empty vector; solid black line), wild-type (GC03; gray line), Cam734 (blue line), Cam734 F144A (red line) or Dd2 (dashed black line). Growth was assessed in the presence of 5 mM CQ in conditions of low Δψ (left panel; pH_external_ 7.20) or high Δψ (right panel; pH_external_ 7.45), as detailed in **Materials and Methods**. Increased growth inhibition correlates with increased CQ accumulation in the yeast cytosol and reflects increased CQR [75]. Δψ increases with increased pH_external_ due to compensatory mechanisms that maintain the electrochemical gradient across the cell membrane. Growth of yeast lines over the pH_external_ range of 7.20–7.45 is surveyed in S6A Fig.

Using quantitative growth rate analyses, we examined the effect of varying external pH (and hence the Δψ) in yeast strains expressing PfCRT isoforms GC03, Cam734, the back-mutant Cam734 F144A (chosen because this back mutation was the most effective at ablating CQ and md-CQ resistance; see Fig 1), or Dd2 (see Table 1 for haplotypes). Growth was assessed in the presence of 5 mM CQ, a concentration required for this drug to exert differential growth inhibitory activity on yeast strains expressing various PfCRT isoforms [22]. As a negative control, we also included yeast harboring no PfCRT (vector control). To examine the effect of Δψ on transport, we assessed growth over a range of pH_external_ values (range of 7.20 [low Δψ] to 7.45 [high Δψ]; S6A Fig). Intriguingly, in low Δψ conditions, growth of yeast expressing Cam734 PfCRT was comparable to that of yeast expressing the CQ-sensitive GC03 PfCRT isoform (Fig 6). However, when the Δψ was clamped to higher values, Cam734 PfCRT conferred a CQR-associated delayed growth phenotype that was intermediate to that of GC03 (wild-type) and Dd2 PfCRT (Fig 6). Of note, the growth phenotype associated with the Cam734 F144A isoform was intermediate to that of empty vector and wild-type PfCRT. This provides evidence that the A144F mutation is critical for drug transport mediated by the Cam734 isoform and is consistent with our drug assay data showing a CQR phenotype for parasites expressing Cam734 *pfcrt* but not the F144A back-mutant (see Fig 1A). These PfCRT-specific phenotypes were not attributable to differences in protein expression, as comparable protein expression of PfCRT variants was observed upon galactose induction of yeast (S6B Fig).

### Differential effects of PfCRT isoforms on parasite digestive vacuole pH and volume

Investigations utilizing the entrapment of a dextran-conjugated NERF to probe the pH and volume of the parasite DV have previously documented the ability of PfCRT mutations to alter DV physiology [40,76,77]. Using similar methods (see **Supplementary Materials and Methods**), we determined the DV pH and volume in isogenic GC03 parasites encoding GC03, Dd2, Cam734 or Cam734 F144A PfCRT, in the presence or absence of CQ concentrations corresponding to twice the CQ 50% lethal dose (LD_50_; see **Supplementary Materials and Methods**). From smallest to largest DV volume, as well as from most alkaline to most acidic DV pH, we observed the order of parasite lines to be: GC03^GC03^, GC03^Cam734 F144A^, GC03^Cam734^, and GC03^Dd2^ (S7 Table). This order was preserved upon a brief (30 min) addition of CQ, which consistently increased DV volume (by 13% to 33%).

## Discussion

To ensure successful progression through their life cycle, drug-resistant *P*. *falciparum* parasites must balance the acquisition of resistance properties with the maintenance of required and often interrelated physiological processes. Focusing on the pathogenic intraerythrocytic stages of parasite growth, we explored herein how novel mutations comprising the unusually polymorphic Cam734 PfCRT variant contribute to this complex relationship. Our analysis of isogenic, *pfcrt*-modified lines reveals that multiple PfCRT mutations possess dual roles, contributing to both quinoline resistance and parasite proliferation. This was most notable for the A144F mutation that is unique to Cam734 PfCRT, which in addition to affecting growth rates proved to be indispensable for parasite resistance to multiple quinoline-type compounds, including CQ, QN, and the first-line ACT partner drug AQ. While these drug IC_50_ shifts are often relatively small, studies have shown that these translate into clear patterns of selection in field parasite populations [7,12]. The pleotropic requirement for the A144F mutation in Cam734 PfCRT-mediated drug resistance is reminiscent of earlier work, in which back-mutation of K76T ablated CQR and nearly halved the degree of parasite resistance to QN [52]. GC03^Cam734 F144A^ parasites appeared CQ-sensitive, but nonetheless showed a 3–fold higher IC_90_ value for md-CQ, believed to be the major driver of selection for mutant *pfcrt* [16], relative to the fully sensitive GC03^GC03^ line (S2 Table). By comparison, GC03^Cam734^ parasites showed a 27–fold md-CQ IC_90_ increase. Similar findings were earlier observed with a PfCRT variant of 7G8 that carries the C350R mutation (the H209 isolate found in French Guiana) [50,60]. This variant was shown to mediate a phenotype of CQ tolerance, which manifested as low CQ IC_50_ values but elevated md-CQ IC_90_ values as well as parasite recrudescence after exposure to CQ concentrations lethal to CQ-sensitive parasites expressing wild-type *pfcrt* [50,60]. We note that parasites encoding Cam734 F144A PfCRT retained K76T as well as 7 other mutations [7]. The clear importance of mutations other than K76T in contributing to CQR can help explain, in areas where novel PfCRT variants have arisen, why the K76T mutation predicts clinical CQR with good sensitivity but only moderate specificity [17,62]. Another important factor driving the reduced specificity of the K76T marker is patient immunity, which in higher-transmission settings of Africa is known to help resolve CQ-resistant infections in CQ-treated patients [78].

Prior studies with asexual blood stage parasites have shown that CQ affects heme disposition (increasing free heme and reducing Hz) and that CQ access to its heme target in the DV is significantly reduced by CQ-resistant mutant forms of PfCRT, which are thought to efflux CQ away from the DV [32,38,52]. We built on these observations by comparing wild-type and variant PfCRT isoforms expressed on the same genetic background. Our results, shown in Figs 4 and 5, provide compelling evidence that the degree of CQ-mediated inhibition of parasite growth is closely correlated with the level of inhibition of Hz formation. Reduced Hz formation was accompanied by the accumulation of reactive free heme, which at high concentrations is presumably the major trigger of parasite death, either alone or conjugated to CQ [7]. Recombinant GC03 parasites expressing the mutant PfCRT Dd2 and Cam734 isoforms (i.e. GC03^Cam734^ and GC03^Dd2^) differed notably from the isogenic clone expressing the wild-type GC03 isoform (GC03^GC03^) in that accumulation of free heme occurred at higher CQ concentrations in the former. Increased free heme was accompanied by lower levels of Hz, consistent with mutant PfCRT being able to efflux drug away from its heme target. Both mutant *pfcrt*-expressing lines also showed reduced levels of heme at low concentrations of CQ or at baseline (no CQ), suggesting a more efficient process of Hz formation under those conditions. The reason for this difference in baseline free heme is not yet known. Evidence suggests that free heme in untreated parasites is sequestered, possibly through association with neutral lipids in the DV [79]. The baseline difference between wild-type and mutant *pfcrt*-expressing parasites may thus be attributed to the larger DV of GC03^Dd2^ and GC03^Cam734^ lines, as compared to GC03^GC03^ parasites (see below and S7 Table), resulting in a lower lipid to aqueous volume ratio. Indeed, a fixed lipid-aqueous portioning coefficient and fixed ratio of lipid to aqueous heme concentration would yield an increased quantity of aqueous free heme (volume × concentration), which in turn would be mostly incorporated into Hz.

With all three lines, levels of undegraded Hb also rose at relatively high CQ fold IC_50_ concentrations, with the highest levels recorded in the GC03^Cam734^ and GC03^Dd2^ lines (see Fig 4C), potentially reflecting increased CQ amounts in the cytosol of these parasites because of higher rates of CQ efflux from the DV. These results expand on the previous observation, in CQ-treated drug-sensitive parasites, that increases in undigested Hb follow significant increases in free heme [32]. This suggests a secondary mode of CQ action, whereby Hb proteolysis is inhibited at higher CQ concentrations, possibly through a physiologic effect of elevated CQ concentrations on Hb endocytosis or the activity of DV-resident hemoglobinases [80,81]. Alternatively, elevated concentrations of free heme in aqueous environments have been shown to form heme aggregates capable of disrupting lipid bilayers and triggering membrane disorder, which in turn could disrupt Hb import and catabolism [82].

Our metabolomics analysis (see Fig 3) and total Hb quantification (see Fig 4C and S5C Fig) reveal major changes in the Hb digestion pathway in the GC03^Cam734^ line when compared with the isogenic lines GC03^Dd2^ and GC03^Dd2^ lines. Given that these changes have been associated with impaired fitness in mutant PfCRT parasites expressing the CQ resistance-conferring Dd2 or 7G8 haplotypes [41], our results raise an obvious question: does Cam734 PfCRT impart a metabolic compensatory mechanism that allows these parasites to circumvent the normally deleterious effects of altered Hb digestion? Previous heterologous expression studies using *Xenopus laevis* oocytes have suggested that mutant PfCRT isoforms might selectively confer transport of the tripeptide glutathione [57], which was earlier proposed to facilitate the degradation of reactive heme and reduce heme-mediated toxicity [36,83]. However, we saw no significant differences in glutathione or any other redox-associated metabolites (see S4 Fig and S6 Table) between isogenic lines encoding Cam734 or Dd2 PfCRT, suggesting that major redox-related metabolic changes are unlikely to account for the improved fitness associated with the Cam734 *pfcrt* allele. In contrast, we observed significant differences in ATP/AMP ratios and central carbon metabolism between lines encoding Cam734 and Dd2 PfCRT (see Fig 3 and S6 Table), implicating changes in energy metabolism as a potential physiologically compensatory mechanism.

Our heterologous yeast expression studies also found that Cam734 is significantly affected by Δψ, resembling a CQ-sensitive PfCRT isoform at low Δψ and a mutant, CQ-resistant PfCRT isoform at high Δψ. This unique plasticity in mediating drug transport may underlie the improved asexual blood-stage fitness associated with the Cam734 PfCRT isoform, as compared with Dd2 (see Fig 2), whereby Cam734-defining mutations confer drug transport only in certain Δψ DV conditions. We also note that CQ transport, as assessed in heterologous expression systems, may only partially account for *in vitro* parasite CQR. This is highlighted by the earlier observation that the CQ-resistant Ecu1110 PfCRT variant (K76T/A220S/N326D/I356L) confers lower parasite CQR, but higher CQ transport, than the related 7G8 PfCRT variant (that in addition carries the C72S mutation) [16,23,77]. The Cam734 isoform might therefore facilitate CQR in part by alleviating CQ-mediated inhibition of an endogenous PfCRT function. Continued elucidation of the elusive function of PfCRT will assist in clarifying these distinctions.

Our physiological studies of isogenic *pfcrt*-modified lines revealed that the DV pH and volume parameters in GC03^Cam734^ parasites were intermediate to GC03^GC03^ and GC03^Dd2^ parasites, consistent with the Cam734 allele producing CQ IC_50_ values between the sensitive wild-type and highly-resistant Dd2 isoforms. In all three isogenic lines, brief exposure to CQ caused DV swelling, with the more CQ-resistant parasites showing the greatest increase in DV volume (S7 Table). As the composition of the DV environment governs the degree of heme-to-Hz conversion [38], we propose that, compared to Dd2 PfCRT, the reduced DV size and more wild-type (GC03) PfCRT-like DV pH associated with the Cam734 isoform might play a role in neutralizing the fitness costs typically associated with mutant PfCRT variants.

Recent evolutionary genetic studies of the adaptive landscapes (i.e. mutational paths and their accompanying fitness costs) associated with drug resistance-conferring mutations in *pfcrt* and *P*. *falciparum* dihydrofolate reductase (*dhfr*) highlight key considerations when hypothesizing how Cam734 *pfcrt* might have evolved: (1) forward *pfcrt* evolution is a physiologically constrained process that is consistent with the rarity of *pfcrt* alleles bearing three or fewer polymorphisms; (2) forward and reverse processes of gene evolution are associated with distinct adaptive landscapes; and (3) adaptive landscapes can be substantially modified by their drug environment [16,84–86]. The spread of CQR in Asia and Africa has long been attributed to a single (Dd2 or Dd2-like) *pfcrt* allele [4]. Of note, Cam734 shares four of the eight mutations comprising both the eight-amino acid Dd2 variant (see Table 1) and the related 6-amino acid variant GB4 (equivalent to Dd2 S326N T356I) found in Africa and also seen in Southeast Asia. Our recent analysis of close to 900 Asian *P*. *falciparum* genomes recently sequenced by the Pf3K consortium [87] estimates the prevalence of Cam734, GB4 and Dd2 *pfcrt* alleles at 15%, 13% and 58% respectively, with the remainder comprising the wild-type allele (3%) and several minor variants. For Cam734, the highest abundance was observed in Cambodia (105 of 570 genomes), Laos (29 of 85) and Vietnam (32 of 97), with a far lower prevalence in Thailand (1 of 148). We posit that, faced with high CQ pressure, parasites underwent mutational bursts (as previously suggested [16]) that led to the evolution of Dd2 *pfcrt*. With reduced CQ pressure, a “reverse” evolutionary process might have led to the loss of some mutations and eventual acquisition of novel ones, as in the case of Cam734 *pfcrt*.

In their report documenting Cam734 *pfcrt* in Cambodia, Durrand *et al*. also reported the related allele Cam738 (akin to Cam734 without the A144F mutation) [47]. We posit that Cam738 served as a mutational precursor of the more evolutionarily successful Cam734 allele. This is supported by the inferior growth of isogenic parasites expressing Cam738 *pfcrt* as compared with Cam734 *pfcrt*, in the absence or presence of CQ or other quinoline drugs (see Figs 1B and 2). Our evidence of reduced growth rates of parasites harboring the Cam738 allele compared with Cam734 is consistent with the absence of Cam738 haplotypes in the recent Pf3K genome data set (https://www.malariagen.net/projects/pf3k). We also note that selective forces favoring mutation of PfCRT residue 144, found herein to be a key mediator of CQR, are apparent in Asia, in some cases requiring two nucleotide substitutions. For example, in the Philippines or in China, PfCRT haplotypes have been detected that, respectively, harbor the mutations A144T or A144Y [88,89]. Interestingly, addition of A144Y to the CQ-resistant Dd2 PfCRT isoform was previously found to abrogate CQ transport in *S*. *cerevisiae* [22].

With sustained exposure to drug selective forces, parasites may evolve intragenic and/or intergenic compensatory changes that allow them to persist even in the absence of drug pressure [90]. The Cam734-defining compensatory mutations identified in our analysis reveal an intragenic basis for the enhanced fitness of this CQ-resistant allele, which could explain its continued presence in Southeast Asia as a minor allele despite the lack of CQ use for several decades to treat *P*. *falciparum* malaria. We note that CQ resistance-conferring mutant *pfcrt* alleles (including Cam734 and Dd2) might also persist in Southeast Asia because of local conditions of decreased genetic diversity and complexity of infections, resulting in less competition among parasite lines, as compared with high-endemicity settings in sub-Saharan Africa, where mutant *pfcrt* alleles are known to rapidly decrease in prevalence in areas without CQ pressure [7].

The degree to which secondary genetic factors also play a role in maintaining mutant *pfcrt* in Southeast Asia is presently unclear. We note that our *pfcrt*-modified lines were generated in GC03 parasites, a clone of the HB3 (Central America) × Dd2 (Asia) genetic cross [91]. These parasites encode the HB3 PfMDR1 haplotype, which differs from Dd2 PfMDR1 at three distinct residues (86, 184, 1042) [92]. A recent study has shown that the PfMDR1 N86Y mutation present in Dd2 augments the degree of CQR imparted by the mutant Dd2 PfCRT isoform [15]. We have observed in prior transfection-based studies that the parasite genetic background dictates the level to which mutant *pfcrt* alleles can mediate CQR [50]. Of note, results obtained herein in GC03 parasites might potentially differ from ones that would be produced with culture-adapted field isolates that naturally harbor the *pfcrt* Cam734 allele. However, to the best of our knowledge, no such isolate has been culture-adapted and reported in the literature. Furthermore, GC03 has been the primary strain used in multiple prior *pfcrt* transfection studies, using either the ZFN method or the earlier approach that used single-site crossovers, thus providing a benchmark against which to assess the current data set [11,16,42,48,50].

The notion that additional genetic changes are required to produce high-level CQR in parasites encoding Cam734 PfCRT evokes a previous finding that mutant PfCRT-encoding parasites can exhibit increased expression of proteins involved in pH regulation, including a V-type H^+^ pyrophosphatase [93]. Our observation that, compared with Dd2, Cam734 PfCRT required a higher Δψ to manifest increased growth in the presence of CQ (consistent with elevated drug transport; see Fig 6) suggests that high-level Cam734 PfCRT-mediated drug resistance may be potentiated by parasite proteins that govern the Δψ across the DV membrane. We speculate that this plasticity in drug transport might be a reflection of the balance that Cam734 PfCRT has achieved in mediating resistance while also avoiding fitness costs. Future genetic dissections of *pfcrt* alleles, as well as candidate secondary genetic modulators (e.g. *pfmdr1*), are possible with the recent advent of efficient parasite ZFN or CRISPR/Cas9–based genome-editing tools [15,94]. Leveraging these approaches with analysis of parasite whole-genome sequences will aid in deciphering the genetic complexities that underlie new and emerging multidrug resistance phenotypes.

## Materials and Methods

### Parasite cultivation and genetic modification

*P*. *falciparum* asexual blood-stage parasites were cultured in human RBCs (Interstate Blood Bank) at 2–4% hematocrit in RPMI-1640-based malaria cell culture medium supplemented with 0.5% Albumax II (Invitrogen) [95]. Cultures were incubated at 37°C in 5% O_2_ / 5% CO_2_ / 90% N_2_. Genetic modification of the parasite *pfcrt* locus is detailed in **Supplementary Materials and Methods** and S1 Fig.

### Drug susceptibility assays

Drug inhibitory concentrations that result in 50% (IC_50_) or 90% (IC_90_) growth inhibition were determined for a panel of drugs (CQ ± 0.8 μM VP, md-CQ, md-AQ, QN, PPQ, LUM, AS, and PND), as described [50]. After 72 h exposure to drug, parasite growth was quantified by staining with SYBR Green I and MitoTracker Deep Red and measuring parasitemia on an Accuri C6 flow cytometer. Reversibility of CQR by 0.8 μM VP was expressed as the CQ RMI, equivalent to the quotient of the CQ+VP IC_50_ divided by the CQ IC_50_ [63]. Statistical significance was determined via non-parametric Mann-Whitney *U* tests using GraphPad Prism 6 software.

### *In vitro* growth assays

As a proxy for *in vitro* fitness, growth of parasite lines was assessed in 1:1 co-culture assays with the fluorescent reporter line NF54^eGFP^, using previously described methods [16]. Briefly, 1:1 co-cultures consisting of the reporter line (GFP^+^) and individual *pfcrt*-modified test lines (GFP^−^) were propagated for 10 generations, and parasitemias were maintained between 0.3% and 8%. The proportion of GFP^−^ parasites was regularly determined by flow cytometric detection of the far-red fluorescent dye SYTO61, which labels the nuclei of infected RBCs (iRBCs), and GFP. Derivation of per-generation selection coefficients (*s*) of test strains is detailed in **Supplementary Materials and Methods**. Statistical significance was assessed via two-way analysis of variance (ANOVA) with Sidak’s post-hoc test using GraphPad Prism 6 software.

### Metabolite extraction and mass spectrometric analysis

All parasite culturing, metabolite extraction, mass spectrometry data acquisition, and data analyses were conducted using previously established methods [41]. Briefly, after double synchronization with 5% sorbitol, late-stage (~36–42 h) *P*. *falciparum* trophozoites were magnetically purified using a SuperMACS magnetic separator (Miltneyi Biotec) and CS columns. Eluted iRBCs were resuspended at 0.4% hematocrit and allowed to recover for 2 h at 37°C in a tissue culture incubator. Cells were then rapidly cooled to 4°C and pelleted by centrifugation at 2,000×g for 5 min. Media was then aspirated away from the iRBC pellets and metabolites were extracted by resuspending cells in cold (4°C) 90% methanol. Samples were homogenized by vortexing and centrifuged at 10,000×g for 5 min at 4°C. The supernatant metabolite extracts were harvested and stored at -80°C until mass spectrometry analysis. Just prior to mass spectrometry, samples were dried under a stream of N_2_ and were resuspended in HPLC-grade water at a 4:1 dilution (relative to the original iRBC pellet volume). High-resolution mass spectrometry data were acquired on a Thermo Fisher Exactive Mass spectrometer in negative mode using 25 min reverse phase gradients and ion-pairing chromatography [41]. Metabolites were identified using the known chromatographic retention times of standards, and metabolite signals were quantified using MAVEN [41]. To allow for more direct metabolite-to-metabolite comparison of phenotypes, raw mass spectrometry signals were expressed as *z*-scores. Briefly, for each metabolite, the mean expected signal ($\bar{x}$) was defined as the mean intensity observed in the control line (GC03^Dd2^). Likewise, the standard deviation (s) for each metabolite signal was calculated from signals observed in the GC03^Dd2^ line (deduced from 3 independent harvests with 4 replicates). The *z*-score (*z*_*i*_) for each observed signal (*x*_*i*_) in test lines was then computed as per the relationship ${\text{z}}_{i}\;=\;[x_{i}-\bar{x}]/\text{s}$ and plotted according to metabolite class. For summary statistics (Fig 3), *z*-scores were calculated from the signals observed for each class. These classes were comprised of metabolites that are directly associated with a metabolic pathway (e.g. TCA metabolism included TCA intermediates as well as the TCA-associated amino acid glutamate). *P* values were computed by one-way ANOVA. All data analyses and statistical tests were conducted using custom in-house software written in R. Metabolite signal intensities are summarized in S5 Table. Metabolite *z*-scores and associated *P* values are found in S6 Table.

### Heme fractionation experiments

The heme fraction profiles of *pfcrt*-modified GC03^GC03^, GC03^Dd2^, and GC03^Cam734^ parasites were determined following recently published and validated protocols [73]. First, parasite growth in response to CQ was determined using the lactate dehydrogenase assay [96]. Heme fractionation assays were then initiated by incubating sorbitol-synchronized, early ring-stage parasites in the absence or presence of CQ in multiples (0.5×, 1×, 2×, 2.5×, and 3×) of the biological CQ IC_50_. After 32 h, iRBCs were treated with 1% saponin to release mature trophozoites, followed by hypotonic lysis and centrifugation. Supernatants were treated with 2% SDS and 2.5% pyridine, yielding the Hb fraction. Pyridine was used as it coordinates to heme forming a monomeric low-spin complex with a distinctive spectrum and is easily detectable by UV-visible spectroscopy, thereby allowing heme species to be quantified. Pellets were treated with 2% SDS and 2.5% pyridine, sonicated, and centrifuged, and supernatants were removed to isolate the free heme fraction. The remaining pellets were solubilized in 2% SDS and 0.1 M NaOH, sonicated, neutralized with HCl, and treated with 2% SDS and 2.5% pyridine to generate the Hz fraction. For each fraction, the UV-visible spectrum of heme present as a heme-pyridine complex was measured with a multi-well plate reader (Spectramax 340 PC, Molecular Devices). The abundance of Hb, free heme, and Hz species was reported as a percent and as an absolute amount of heme Fe per cell. Parasites were quantified using flow cytometry, as previously described [73]. Statistical significance was assessed via unpaired *t* tests with Welch’s correction using GraphPad Prism 6 software.

### Yeast drug transport assays

Cultivation, transfection, and quantitative growth rate analysis of *S*. *cerevisiae* yeast strains employed previously detailed protocols [22,75]. Briefly, CH1305 yeast strains were transfected with either pYES2 (blank vector) or pYES2-derived plasmids encoding the galactose/raffinose-inducible, codon-optimized PfCRT isoforms GC03 (HB3; wild-type), Cam734, Cam734 F144A (also known as Cam738), or Dd2. Quantitative assessment of yeast growth, a validated proxy for CQ transport [74], was performed in PfCRT-inducing (galactose/raffinose) or PfCRT-noninducing (glucose) conditions, with a starting cell density (OD_600_) of 0.1. Yeast growth ± 5 mM CQ was measured in triplicate with a Tecan GENios microplate reader following established parameters [22]. PfCRT protein expression of yeast lines was evaluated using Western blot analysis, demonstrating comparable protein levels across all lines (see **Supplementary Materials and Methods** and S6B Fig).

## Supporting Information

S1 Text Supplementary Materials and Methods. (DOCX)

S1 Fig Genetic modification of the *pfcrt* locus via zinc-finger nucleases (ZFNs). **(A)** Genetic engineering strategy. Briefly, parasites were transfected with a donor template plasmid (p*crt*-h*dhfr*) with the coding region corresponding to exons 2 to 13 (e2-13) of a *pfcrt* allele of interest (indicated in purple; see Table 1 for full list of *pfcrt* alleles). Donor plasmids also include the following: a *P*. *berghei crt* (*pbcrt*) 3′ untranslated region (UTR) sequence, a human *dhfr* (h*dhfr*) selection cassette, and flanking left (~0.4 kb upstream of the intron 1-exon 2 boundary) and right (~1 kb native 3′ UTR) homology regions [48]. Donor plasmid-enriched parasites were transfected with pZFN^*crt*^-*bsd*, which encodes a pair of *pfcrt*-specific ZFNs (ZFN_L_ and ZFN_R_) via the *calmodulin* (*cam*) promoter and includes the *blasticidin S deaminase* (*bsd*) selection cassette. Operating as an obligate heterodimer, ZFN_L_ and ZFN_R_ catalyze a double-stranded DNA break in the *pfcrt* intron 1-exon 2 region (indicated with a red bolt). Recombinant parasites that were successfully generated via DNA repair mechanisms encode *pfcrt* exon 1 as well as the e2-13 sequence bearing mutations of interest. Recombinant parasites were cloned by limiting dilution, their genetic editing verified by diagnostic PCR (see **S1B Fig**), and sequence integrity verified at the DNA and RNA levels. **(B)** Diagnostic PCRs of representative recombinant parasites encoding full-length (GC03^Cam734^) and back-mutated (GC03^Cam734 F144A^) *pfcrt* alleles. Controls include genetically unedited parental parasites (GC03), unedited donor plasmid-enriched parasites (GC03 + either p*crt* ^Cam734^-h*dhfr* or p*crt* ^Cam734 F144A^-h*dhfr*) and donor plasmids alone (p*crt* ^Cam734^-h*dhfr* or pcrt^Cam734 F144A^-h*dhfr*). Primer (p) locations are illustrated in **S1A Fig**. PCR amplicons for ZFN-edited lines demonstrated the expected sizes of 0.4 kb (p5+p6), 1.2 kb (p7+p6), 2.5 kb (p8+p9), and 2.7 kb (p7+p10). **(C)** Western blot analysis of isogenic *pfcrt*-modified parasite lines showing equivalent PfCRT protein expression levels, as detected using anti-PfCRT primary antibodies that identify the ~42 kDa protein as expected [10]. Antibodies to the ~25 kDa PfERD2 protein were used as a reference. We note that our recent study by Gabryszewski *et al*. [16], which employed the identical ZFN approach used herein, examined PfCRT protein expression levels in seven *pfcrt*-modified lines. Results showed no more than a 10% difference on average between any two given lines, with none of these differences being statistically significant. There was also no correlation between PfCRT protein levels and either the degree of CQ resistance or the rates of *in vitro* parasite growth. In earlier studies with the former single-site crossover method, we compared protein expression levels in a panel of *pfcrt*-modified lines, which also showed no significant difference between lines and no correlation with CQ IC_50_ values [11,42,50,52]. Of note, the new ZFN method has the major benefit that it does not result in an appreciable reduction in protein expression levels, unlike the earlier single-site crossover method that retained downstream partial gene fragments and showed lower levels of expression of the full-length recombinant protein compared to non-modified lines.
(EPS)

S2 Fig Parasite responses to lumefantrine, artesunate, and pyronaridine. Following 72 h exposure of parasites to the indicated antimalarial drugs, parasite growth was assessed using flow cytometry, as detailed in **Materials and Methods**. Bar graphs correspond to mean ± SEM IC_50_ values. Results encompass 2 to 12 independent assays conducted in duplicate. Statistical differences were determined via non-parametric Mann-Whitney *U* tests, using the mean IC_50_ value of Cam734 *pfcrt*-expressing GC03^Cam734^ parasites as the comparator. IC_50_ and IC_90_ values and Hill slopes are summarized along with corresponding statistical tests in S2 Table. *P<0.05; **P<0.01; ***P<0.001. ****P<0.0001.
(EPS)

S3 Fig *In vitro* growth plots of *pfcrt*-modified and reference parasites. Co-cultures consisting of 1:1 proportions of individual GFP-negative (GFP^−^) test lines and a GFP-positive (GFP^*+*^) reporter line were seeded at day 0 and regularly monitored by flow cytometry for 10 parasite generations (see Materials and Methods). Three independent assays were conducted in duplicate in the absence or presence of a sub-lethal dose of CQ (7.5 nM, equivalent to ~0.5× CQ IC_50_ of the CQ-sensitive line GC03^GC03^). **(A)** Plots of the mean ± SEM proportion of GFP^−^ test lines (*p*) as a function of number of parasite generations (*t*). GFP^+^ (reporter line only) and GFP^−^ (GC03^GC03^ only) control (ctrl) lines exhibited steady fluorescence levels over the duration of the experiment. **(B)** Plots of mean ± SEM natural log (ln)-transformed ratios of the proportion of a GFP^−^ test line to the GFP^+^ reporter line at time *t* (*p*_t_/*q*_t_). This parameter was used to derive *in vitro* growth selection coefficients (*s*), as detailed in **Supplementary Materials and Methods**.
(EPS)

S4 Fig Individual metabolite profiles of isogenic, mutant *pfcrt*-expressing parasites. Metabolite extracts were derived from tightly synchronized trophozoite-stage isogenic (GC03) parasites encoding either Dd2 or Cam734 *pfcrt* and analyzed by mass spectrometry. Metabolite signals were converted to *z*-scores (see Materials and Methods). Each bar shows the *z*-score for an experimental replicate obtained for GC03^Dd2^ (black) or GC03^Cam734^ (red) parasites. Dashed lines represent lower (5%) and upper (95%) boundaries for the normal distribution, as defined for GC03^Dd2^ parasites. Metabolites were harvested on three independent occasions (*n* = 4 to 6 total replicates per parasite strain). Metabolite *z*-scores, *P* values, and full names of individual metabolites are detailed in S6 Table. TCA, tricarboxylic acid; PPP, pentose phosphate pathway; Nt, nucleotide; AA, amino acid; NAc, N-Acetylated.
(EPS)

S5 Fig Absolute concentrations of heme species in control and chloroquine (CQ)-treated *pfcrt*-modified parasite lines. The amount in femtograms (fg) of heme Fe per cell present as **(A)** free heme, **(B)** hemozoin (Hz), or **(C)** hemoglobin (Hb) was measured spectrophotometrically in isogenic parasites expressing the wild-type (CQ-sensitive) GC03 *pfcrt* allele or mutant (CQ-resistant) Dd2 and Cam734 *pfcrt* alleles. Prior to heme fractionation, synchronous parasites were exposed for 32 h to CQ concentrations corresponding to line-specific CQ IC_50_ folds (1× CQ IC_50_ values of 19.5 nM, 187 nM, and 90.9 nM for GC03^GC03^, GC03^Dd2^, and GC03^Cam734^, respectively. In absolute values, 0.5× CQ IC_50_ for GC03^Cam734^ is equivalent to 2.3× the CQ IC_50_ for GC03^GC03^ and 0.25× the CQ IC_50_ for GC03^Dd2^). Bar graphs indicate mean ± SEM percentage values for 5 to 8 technically independent replicates. For each parasite line, values obtained for CQ-treated samples (gray bars) were compared against the untreated control (black bars), and statistical significance was determined via unpaired *t* tests with Welch’s correction. *P<0.05; **P<0.01; ***P<0.001. ****P<0.0001.
(EPS)

S6 Fig Membrane potential (Δψ) dependence of PfCRT-mediated yeast growth delay and PfCRT isoform expression. **(A)** Growth delay of yeast lines encoding wild-type (gray diamonds), Dd2 (black squares), Cam734 (blue triangles), or Cam734 F144A (red triangles, also known as Cam738) PfCRT was measured in the presence of 5 mM CQ and normalized to growth of yeast harboring empty vector (see Materials and Methods). These PfCRT isoforms were tagged to a V5 epitope at their C terminus [75]) Growth was examined for a range (7.20 to 7.45) of pH_external_ values. Through compensatory mechanisms, the yeast Δψ increases with increased pH_external_. CQ transport rates were calculated as previously described [75]. Error bars indicate the SEM for at least three independent yeast clones analyzed in triplicate. **(B)** Yeast protein extracts were subjected to Western blot analysis with anti-V5 antibodies, as described in **Supplementary Materials and Methods**. Briefly, total protein concentrations in the yeast crude membrane fractions were quantified using an amido black assay and 7.0 μg of protein per sample was electrophoretically separated and transferred onto a PVDF membrane. Levels of the 51.8-kDa PfCRT-V5 protein were comparable for all PfCRT isoforms (GC03, Dd2, Cam734, and Cam734 F144A). A yeast strain harboring an empty vector was included in the analysis as a negative control.
(EPS)

S1 Table Primers used in this study. Nucleotides corresponding to restriction sites are underlined. F, forward; R, reverse; gDNA, genomic DNA; *pbcrt*, *P*. *berghei* chloroquine resistance transporter; UTR, untranslated region.
(PDF)

S2 Table Antimalarial IC_50_ and IC_90_ values of *pfcrt*-modified and reference parasite lines. IC_50_ and IC_90_ values (nM) indicate the mean ± SEM, as determined in 2 to 12 independent assays performed in duplicate. CQ + VP assays were performed with 0.8 μM VP. CQ, chloroquine; VP, verapamil; md-CQ, monodesethyl-chloroquine; md-AQ, monodesethyl-amodiaquine; QN, quinine; PPQ, piperaquine; LUM, lumefantrine; AS, artesunate; PND, pyronaridine; *n*, number of assays. *P* values were determined in a non-parametric Mann-Whitney *U* test versus the parasite line GC03^Cam734^. *P* values <0.05 are indicated in bold and shaded in gray. Hill slopes were calculated from the dose-response data using GraphPad Prism 6 software.
(PDF)

S3 Table Verapamil-mediated CQ resistance reversibility of *pfcrt*-modified and reference parasite lines. Reversibility of chloroquine (CQ) resistance by 0.8 μM verapamil (VP) is indicated as the CQ response modification index (RMI), equivalent to (IC_50_ for CQ+VP) ÷ (IC_50_ for CQ only). Shown are mean RMI ± SEM values, as determined in 5 to 12 independent assays. *n*, number of assays. *P* values were determined in a non-parametric Mann-Whitney *U* test versus the parasite line GC03^Cam734^. *P* values <0.05 are indicated in bold and shaded in gray.
(PDF)

S4 Table *In vitro* growth selection coefficients of *pfcrt*-modified and reference parasite lines. Parasite *in vitro* growth was evaluated in the absence or presence of a sub-lethal dose of CQ (7.5 nM; ~0.5× CQ IC_50_ of the CQ-sensitive reference line GC03^GC03^) and normalized against GC03^Cam734^ in the absence of drug pressure (6 total replicates per condition). As detailed in **Materials and Methods**, the per-generation selection coefficient (indicated above as *s* ± SEM) was derived from the relative fitness index (*ω'*) as per the relationship *s = ω'*– 1, such that *s*<0 and *s*>0 respectively indicate growth inferior or superior to the GC03^Cam734^ parasite line, which encodes the full-length Cam734 *pfcrt* allele. CQ, chloroquine; *P*_*1*_, *P* value for inter-strain comparisons, determined versus the parasite line GC03^Cam734^ using two-way ANOVA with Sidak’s post-hoc test; *P*_*2*_, *P* value for intra-strain comparisons, determined for a given parasite strain in the absence versus presence of 7.5 nM CQ using two-way ANOVA with Sidak’s post-hoc test. *P* values <0.05 are indicated in bold and shaded in gray.
(PDF)

S5 Table Metabolite mass spectroscopy signal intensities. Mass spectrometric signal intensities for metabolites derived from trophozoite-stage isogenic (GC03) parasites encoding either Dd2 or Cam734 *pfcrt*, as detailed in **Materials and Methods**. Metabolites were harvested on three independent occasions, yielding 4 and 6 individual replicates (r) for the GC03^Dd2^ and GC03^Cam734^ strains, respectively.
(PDF)

S6 Table Metabolite *z*-scores and *P* values. Metabolite classes and individual metabolite *z*-scores (see Materials and Methods for *z*-score derivation). *P* values were determined using one-way ANOVA. Corresponding metabolite signal intensities are reported in S5 Table. Metabolites were harvested on three independent occasions, yielding 4 and 6 individual replicates (r) for the GC03^Dd2^ and GC03^Cam734^ strains, respectively. *P* values <0.05 are shaded in gray.
(PDF)

S7 Table Digestive vacuole volume size and pH of *pfcrt*-modified lines. For the indicated isogenic, *pfcrt*-modified lines, digestive vacuole (DV) volume size and pH were determined using spinning disk confocal microscopy and single-cell photometry, respectively, as detailed in **Supplementary Materials and Methods**. Measurements were made following 30 min exposure to no drug or 2× CQ LD_50_. Results are reported as mean ± SEM DV volume size (μm^3^) or pH, as determined for ≥20 parasites, beginning in each case with tightly synchronized young trophozoites. DV volume values for CQ-treated parasites were compared against those of untreated controls to determine the percent increase in size.
(PDF)
