# Supplementary material for: Evolution of Fitness Cost-Neutral Mutant PfCRT Conferring P. falciparum 4-Aminoquinoline Drug Resistance Is Accompanied by Altered Parasite Metabolism and Digestive Vacuole Physiology
Source: PLoS Pathog. 2016 Nov 10;12(11):e1005976. doi: 10.1371/journal.ppat.1005976 (PMC5104409; doi:10.1371/journal.ppat.1005976)
Supplement: S1 Text — (DOCX) [file ppat.1005976.s001.docx]

**S1 Text: Supplementary Materials and Methods**

**Genetic modification of the parasite *pfcrt* locus.** Plasmids encoding a set of full-length (e.g. Cam734) or back-mutated (e.g. Cam734 F144A) *pfcrt* alleles were used as templates for PCR amplification of *pfcrt* exons 2 through 13 with primers p1 + p2 (see **S1 Table** for all primer details). To generate *pfcrt* donor transfection plasmids, these amplicons were cloned into the p*crt*^Dd2^-h*dhfr* plasmid backbone [1] using NcoI and SalI restriction sites.

A schematic of our genetic engineering strategy is shown in **S1A Fig**. Briefly, GC03 parasites were transfected with 40 μg donor plasmid (p*crt*-h*dhfr*) and selected with 2.5 nM WR99210 (Jacobus Pharmaceuticals). Donor plasmid-enriched parasites were subsequently transfected with 40 μg pZFN*^crt^*-*bsd* and pressured with 2.5 nM WR99210 and 2 μg/ml Blasticidin HCl (Invitrogen) for six days, followed by prolonged pressure with 2.5 nM WR99210. To screen for desired genetic modification events (see **S1B Fig**), we analyzed recombinant parasites by direct blood PCR (KAPA Biosystems), as earlier described [2]. Bulk cultures demonstrating evidence of genetic editing were then cloned by limiting dilution [3] and two parasite clones per successful editing were selected for further analysis. The previously generated GC03^Dd2^ line [1], as well as the unedited GC03 parental line, were also included in subsequent analyses. To verify sequence integrity of recombinant parasite clones, the *pfcrt* locus was PCR-amplified using primers p10 + p7 and subsequently sequenced with primers p3 and p4. Sequence integrity was also verified at the transcript level by RT-PCR, and cDNA fully sequenced to ensure error-free editing, as previously described [2]. We note that our initial study that described *pfcrt*-specific zinc-finger nucleases (ZFNs) used whole-genome sequence analysis on edited lines, which confirmed no off-target activity [1]. That finding is consistent with the absence of non-homologous end-joining processes in this parasite and the dependence on homology-directed recombination [4], which in this case specifically targets *pfcrt*.

Trophozoite-stage parasites were doubly synchronized with 5% sorbitol and lysed with 0.1% saponin. Extracts from three independent 2-ml cultures (~4x10^7^) were pooled (~10^8^ parasites total) and lysed with 0.1% saponin. The resulting lysates were washed with PBS, resuspended in RIPA Lysis Buffer (Boston BioProducts) supplemented with Halt Protease Inhibitor Cocktail (ThermoFisher Scientific), and stored at -80°C. Thawed proteins extracts were incubated at 65°C for 10 min in sample buffer and subjected to a 4-12% Criterion XT Bis-Tris Gel (BioRad). These were subsequently transferred onto a Nitrocellulose membrane (Millipore). Antibody incubations were as follows: rabbit polyclonal anti-PfCRT [5] or anti-PfERD2 (MRA-1; MR4, BEI Resources, NIAID, NIH) primary antibodies, and anti-rabbit horseradish peroxidase-conjugated secondary antibody. Proteins were visualized by chemiluminescence and the images captured using Image Studio software (LI-COR). Results are shown in **S1C Fig**.

**Derivation of growth selection coefficients.** *In vitro* growth of parasite co-cultures was determined every 2–3 days for 10 generations, as described in **Materials and Methods** and further detailed elsewhere [2]. The ratio of the frequencies of the GFP^–^ test strain (*p_t_*) and the GFP^+^ reporter strain (*q_t_*) at time *t* was natural log-transformed and used to calculate the fitness (*ω*) of the test allele as per the relationship $\ln\left( \frac{p_{t}}{q_{t}} \right)=\ln\left( \frac{p_{0}}{q_{0}} \right)+tln(\omega)$ [2]. The relative fitness for each test strain was normalized to that of the GC03^Cam734^ parasite line, which encodes the full-length Cam734 *pfcrt* allele, and was expressed as the normalized relative fitness (*ω*′). The per-generation selection coefficient (*s*) for each test strain was subsequently computed as per the relationship *s* = *ω*′ – 1 [6]. Statistical significance was assessed via two-way ANOVA with Sidak’s post-hoc test using GraphPad Prism 6 software.

**Comparison of protein levels of yeast-expressed PfCRT isoforms.** Protein extracts from yeast strains harboring V5-tagged PfCRT isoforms or empty vector were prepared and subjected to Western blot analysis, as in previous studies [7]. Briefly, total protein in the yeast crude membrane fraction was quantified using the Amido Black assay. 7 µg total protein was subsequently incubated at 65°C for 5 min, electrophoretically separated on a 12% SDS-PAGE gel, and transferred onto a PVDF membrane. Membranes were incubated with horseradish peroxidase-conjugated anti-V5-antibodies, and the 51.8-kDa PfCRT-V5 protein was detected by chemiluminescence using Amersham Hyperfilm ECL.

**Spinning disk confocal microscopy (SDCM).** Measurements of parasite digestive vacuole (DV) volumes, data acquisition, deconvolution, and 3D restoration were performed as previously described [8-10] using a customized Perkin-Elmer spinning disk confocal microscope. Images were acquired with a 491 nm laser line at 200 ms exposure and 35% laser power. For CQ treatment, cultures were treated for 30 min in malaria culture media with 0.5% Albumax containing two times the 50% lethal dose of CQ (2× CQ LD_50,_ corresponding to 200 nM for GC03^GC03^ and GC03^Cam734 F144A^; 1.2 μM for GC03^Cam734^; and 2.2 μM for GC03^Dd2^, as determined using established protocols [11]. These concentrations collectively span the range of 12×–18× CQ IC_50_; see **S2 Table**). Cells were subsequently mounted onto a coverslip in HBSS containing the same concentration of CQ, and images were obtained.

**Single-cell photometry (SCP).** SCP experiments were done as previously detailed [12,13] using a custom system comprised of a Nikon Diaphot microscope, a Photometrics Sensys 12-bit CCD camera, associated optics, custom perfusion cells, and custom dynamic thresholding software. Parasite cultures were treated for 30 min with media with 0.5% Albumax and a concentration of CQ corresponding to 2× CQ LD_50_ of each line (as detailed for SDCM experiments above). To measure pH, parasites were perfused with Hanks’ balanced salt solution (HBSS) with the same CQ concentration, followed by calibration solutions.

**Supplementary References**

1. Straimer J, Lee MC, Lee AH, Zeitler B, Williams AE, et al. Site-specific genome editing in *Plasmodium falciparum* using engineered zinc-finger nucleases. Nat Methods. 2012; 9(10):993-8. doi: 10.1038/nmeth.2143. PMID: 22922501.

2. Gabryszewski SJ, Modchang C, Musset L, Chookajorn T, Fidock DA. Combinatorial genetic modeling of *pfcrt*-mediated drug resistance evolution in *Plasmodium falciparum*. Mol Biol Evol. 2016; 33(6):1554-70. doi: 10.1093/molbev/msw037. PMID: 26908582.

3. Adjalley SH, Johnston GL, Li T, Eastman RT, Ekland EH, et al. Quantitative assessment of *Plasmodium falciparum* sexual development reveals potent transmission-blocking activity by methylene blue. Proc Natl Acad Sci U S A. 2011; 108(47):E1214-23. doi: 10.1073/pnas.1112037108. PMID: 22042867.

4. Lee AH, Symington LS, Fidock DA. DNA repair mechanisms and their biological roles in the malaria parasite *Plasmodium falciparum*. Microbiol Mol Biol Rev. 2014; 78(3):469-86. doi: 10.1128/MMBR.00059-13. PMID: 25184562.

5. Fidock DA, Nomura T, Talley AK, Cooper RA, Dzekunov SM, et al. Mutations in the *P. falciparum* digestive vacuole transmembrane protein PfCRT and evidence for their role in chloroquine resistance. Mol Cell. 2000; 6(4):861-71. PMID: 11090624.

6. Baker S, Duy PT, Nga TV, Dung TT, Phat VV, et al. Fitness benefits in fluoroquinolone-resistant *Salmonella* Typhi in the absence of antimicrobial pressure. Elife. 2013; 2:e01229. doi: 10.7554/eLife.01229. PMID: 24327559.

7. Baro NK, Pooput C, Roepe PD. Analysis of chloroquine resistance transporter (CRT) isoforms and orthologues in *S. cerevisiae* yeast. Biochemistry. 2011; 50(31):6701-10. doi: 10.1021/bi200922g. PMID: 21744797.

8. Gaviria D, Paguio MF, Turnbull LB, Tan A, Siriwardana A, et al. A process similar to autophagy is associated with cytocidal chloroquine resistance in *Plasmodium falciparum*. PLoS One. 2013; 8(11):e79059. doi: 10.1371/journal.pone.0079059. PMID: 24278114.

9. Gligorijevic B, Bennett T, McAllister R, Urbach JS, Roepe PD. Spinning disk confocal microscopy of live, intraerythrocytic malarial parasites. 2. Altered vacuolar volume regulation in drug resistant malaria. Biochemistry. 2006; 45(41):12411-23. doi: 10.1021/bi0610348. PMID: 17029397.

10. Gligorijevic B, McAllister R, Urbach JS, Roepe PD. Spinning disk confocal microscopy of live, intraerythrocytic malarial parasites. 1. Quantification of hemozoin development for drug sensitive versus resistant malaria. Biochemistry. 2006; 45(41):12400-10. doi: 10.1021/bi061033f. PMID: 17029396.

11. Sherlach KS, Roepe PD. Determination of the cytostatic and cytocidal activities of antimalarial compounds and their combination interactions. Curr Protoc Chem Biol. 2014; 6(4):237-48. doi: 10.1002/9780470559277.ch140125. PMID: 25445179.

12. Bennett TN, Kosar AD, Ursos LM, Dzekunov S, Singh Sidhu AB, et al. Drug resistance-associated PfCRT mutations confer decreased *Plasmodium falciparum* digestive vacuolar pH. Mol Biochem Parasitol. 2004; 133(1):99-114. PMID: 14668017.

13. Dzekunov SM, Ursos LM, Roepe PD. Digestive vacuolar pH of intact intraerythrocytic *P. falciparum* either sensitive or resistant to chloroquine. Mol Biochem Parasitol. 2000; 110(1):107-24. PMID: 10989149.
