# Supplementary material for: Evolution of Fitness Cost-Neutral Mutant PfCRT Conferring P. falciparum 4-Aminoquinoline Drug Resistance Is Accompanied by Altered Parasite Metabolism and Digestive Vacuole Physiology
Source: PLoS Pathog. 2016 Nov 10;12(11):e1005976. doi: 10.1371/journal.ppat.1005976 (PMC5104409; doi:10.1371/journal.ppat.1005976)
Supplement: S1 Table — Nucleotides corresponding to restriction sites are underlined. F, forward; R, reverse; gDNA, genomic DNA; pbcrt, P. berghei chloroquine resistance transporter; UTR, untranslated region. (PDF) [file ppat.1005976.s008.pdf]

S1 Table. Primers used in this study.

Primer	Oligonucleotide sequence (5’ → 3’)	Description	Lab name
p1	AACCATGGATTTATTGTGTAATAATTGAATCGACG	NcoI-*pfcrt* exon 13 R	p1640
p2	CCCTTGTCGACCTTAACAGATGGCTC	SalI-*pfcrt* intron1-exon 2 F	p3519
p3	TCAAACATGACAAGGGAAATAGT	*pfcrt* exon 5 F	p2427
p4	CCAAGAATAAACATGCGAAACC	*pfcrt* exon 7 R	p3806
p5	CTTGAATTCGACCTTAACAGATGGCTCAC	*pfcrt* exon 2 F	p3264
p6	CTTATCGATAAGCAGAAGAACATATTAATAGGAATACTTAATTG	*pfcrt* exon 3 R	p3265
p7	CTTGGGCCCAAGTTGTACTGCTTCTAAGC	*pfcrt* gDNA 5′ UTR F	p3404
p8	CTCGAGATGGTTGGTTCGCTAAACTGC	h*dhfr* F	p3315
p9	TTGACCCTTATATATTCCACCCA	*pfcrt* gDNA 3′ UTR R	p3403
p10	GAGGCGCCTATTTCAAAAATCTTAGCATAAGGATT	*pbcrt* 3′ UTR R	p1644

Nucleotides corresponding to restriction sites are underlined. F, forward; R, reverse; h*dhfr*, human dihydrofolate reductase; gDNA, genomic DNA; *pbcrt, P. berghei chloroquine resistance transporter*; UTR, untranslated region.
