# Supplementary material for: Evolution of Fitness Cost-Neutral Mutant PfCRT Conferring P. falciparum 4-Aminoquinoline Drug Resistance Is Accompanied by Altered Parasite Metabolism and Digestive Vacuole Physiology
Source: PLoS Pathog. 2016 Nov 10;12(11):e1005976. doi: 10.1371/journal.ppat.1005976 (PMC5104409; doi:10.1371/journal.ppat.1005976)
Supplement: S2 Table — IC50 and IC90 values (nM) indicate the mean ± SEM, as determined in 2 to 12 independent assays performed in duplicate. CQ + VP assays were performed with 0.8 μM VP. CQ, chloroquine; VP, verapamil; md-CQ, monodesethyl-chloroquine; md-AQ, monodesethyl-amodiaquine; QN, quinine; PPQ, piperaquine; LUM, lumefantrine; AS, artesunate; PND, pyronaridine; n, number of assays. P values were determined in a non-parametric Mann-Whitney U test versus the parasite line GC03Cam734. P values <0.05 are indicated in bold and shaded in gray. Hill slopes were calculated from the dose-response data using GraphPad Prism 6 software. (PDF) [file ppat.1005976.s009.pdf]

S2 Table. Antimalarial IC_{50} and IC_{90} values of *pfcrt*-modified and reference parasite lines (page 1 of 3).

	GC03^Cam734 back-mutants									
	$GC03^{Cam734}$	D75N	F144A	I148L	T194I	S333T	$GC03^{GC03}$	$GC03^{Dd2}$	GC03	Dd2
CQ IC_{50}	73.4 ± 5.2	42.8 ± 4.7	11.4 ± 1.1	34.2 ± 3.3	56.1 ± 3.7	38.4 ± 4.5	14.0 ± 1.6	189 ± 19.2	11.8 ± 0.7	217 ± 16.7
n	12	6	6	5	5	6	10	10	5	5
P	–	**0.002**	**0.0001**	**0.0006**	0.06	**0.0008**	**< 0.0001**	**< 0.0001**	**0.0003**	**0.0003**
CQ IC_{90}	148 ± 10.1	91.4 ± 9.0	27.8 ± 2.2	87.2 ± 3.9	114 ± 7.1	81 ± 9.3	23.1 ± 2.1	329 ± 25.6	20.9 ± 2.2	412 ± 30
n	12	6	6	5	5	6	10	9	5	5
P	–	**0.002**	**0.0001**	**0.0003**	0.08	**0.0004**	**< 0.0001**	**< 0.0001**	**0.0003**	**0.0003**
Hill Slopes	3.4 ± 0.1	3.3 ± 0.1	3.1 ± 0.2	2.9 ± 0.2	3.5 ± 0.2	3.2 ± 0.2	5.6 ± 0.4	3.2 ± 0.3	5.8 ± 0.3	4.9 ± 0.1
CQ+VP IC_{50}	40.5 ± 4.0	41.1 ± 8.4	10.8 ± 2.6	31.2 ± 1.9	33.2 ± 3.0	23.4 ± 6.0	18.0 ± 2.1	29.2 ± 4.6	14.8 ± 2.9	68.7 ± 10.2
n	12	6	6	5	5	6	10	10	5	5
P	–	0.75	**0.002**	0.08	0.15	**0.024**	**0.0011**	**0.05**	**0.006**	**0.027**
CQ+VP IC_{90}	103 ± 9.2	86.4 ± 16.5	30.8 ± 7.8	89.8 ± 9.7	88.3 ± 7.9	63.6 ± 15.7	31.9 ± 4.5	110 ± 13.7	23.2 ± 4.5	156 ± 14.1
n	12	6	6	5	5	6	10	10	5	5
P	–	0.58	**0.0013**	0.15	0.12	**0.032**	**0.0002**	0.67	**0.002**	**0.02**
Hill Slopes	2.7 ± 0.1	2.9 ± 0.1	3.2 ± 0.2	2.7 ± 0.2	2.8 ± 0.2	2.8 ± 0.2	5.6 ± 0.3	2.3 ± 0.1	5.9 ± 0.4	3.7 ± 0.3
md-CQ IC_{50}	489 ± 36.1	320 ± 26.5	31.9 ± 3.5	131 ± 17.7	404 ± 15.2	317 ± 27.2	18.6 ± 0.8	804 ± 60.2	20.4 ± 1.6	948 ± 83.2
n	12	6	6	5	5	6	10	10	5	5
P	–	**0.003**	**0.0001**	**0.0003**	0.1	**0.007**	**< 0.0001**	**< 0.0001**	**0.0003**	**0.0003**
md-CQ IC_{90}	925 ± 66.7	653 ± 74.5	104 ± 4.6	301 ± 34.6	823 ± 38.1	650 ± 68.1	34.6 ± 1.6	1582 ± 94	36.9 ± 4.0	1522 ± 100
n	12	6	6	5	5	6	10	10	5	5
P	–	**0.007**	**0.0001**	**0.0003**	0.18	**0.007**	**< 0.0001**	**< 0.0001**	**0.0003**	**0.002**
Hill Slopes	3.1 ± 0.1	3.1 ± 0.1	2.3 ± 0.1	2.7 ± 0.2	3.0 ± 0.1	3.1 ± 0.1	3.5 ± 0.3	3.6 ± 0.2	4.7 ± 0.5	4.3 ± 0.6
md-AQ IC_{50}	61.1 ± 3.1	40.9 ± 2.1	15.2 ± 1.6	27.0 ± 1.0	50.9 ± 3.1	42.5 ± 3.8	14.6 ± 1.3	58.8 ± 3.7	13.8 ± 1.5	66.9 ± 7.4
n	12	6	6	5	5	6	10	10	5	5
P	–	**0.0004**	**0.0001**	**0.0003**	0.08	**0.003**	**< 0.0001**	0.75	**0.0003**	0.57
md-AQ IC_{90}	107 ± 4.4	67.9 ± 6.0	24.8 ± 2.3	52.4 ± 2.4	92.0 ± 9.0	72.9 ± 8.2	21.4 ± 2.2	105 ± 7.1	20.1 ± 3.3	111 ± 5.5
n	12	6	6	5	5	6	10	10	5	5
P	–	**0.0008**	**0.0001**	**0.0003**	0.08	**0.003**	**< 0.0001**	0.53	**0.0003**	0.57
Hill Slopes	4.0 ± 0.2	4.6 ± 0.3	5.0 ± 0.3	3.7 ± 0.2	4.3 ± 0.1	4.4 ± 0.3	9.0 ± 0.7	4.3 ± 0.4	7.4 ± 1.4	5.5 ± 0.5

S2 Table (page 2 of 3).

	$GC03^{Cam734}$	$GC03^{Cam734}$ back-mutants D75N	F144A	I148L	T194I	S333T	$GC03^{GC03}$	$GC03^{Dd2}$	GC03	Dd2
QN IC_{50}	37.2 ± 4.2	39.7 ± 5.8	23.9 ± 2.3	26.8 ± 3.4	36.4 ± 1.8	37.4 ± 4.8	55.1 ± 4.6	39.3 ± 4.2	65.4 ± 11.5	91.7 ± 11.7
n	10	6	6	5	3	6	10	10	3	3
P	–	0.71	**0.011**	0.1	0.81	0.86	**0.0052**	0.52	**0.028**	**0.007**
QN IC_{90}	144 ±11.0	114 ± 13.1	77.9 ± 6.7	120 ±15.8	114 ± 12.4	108 ± 10.0	171 ± 6.5	157 ± 11.6	173 ± 6.3	290 ± 52.5
n	10	6	6	5	3	6	10	10	3	3
P	–	0.11	**0.001**	0.2	0.24	0.07	0.09	0.43	0.16	**0.014**
Hill Slopes	1.9 ± 0.1	2.3 ± 0.1	2.3 ± 0.1	1.8 ± 0.1	2.1 ± 0.1	2.2 ± 0.2	2.3 ± 0.1	1.9 ± 0.1	2.2 ± 0.3	2.2 ± 0.1
PPQ IC_{50}	9.1 ± 0.6	11.9 ±1.2	11.1 ± 0.6	14.1 ± 1.5	9.8 ± 1.2	9.8 ± 0.5	11.1 ± 0.5	13.3 ± 1.1	10.4 ± 0.6	12.2 ± 0.7
n	8	6	6	3	3	6	8	8	3	3
P	–	0.11	0.08	**0.049**	0.38	0.49	0.1	**0.007**	0.5	**0.049**
PPQ IC_{90}	21.4 ± 1.9	25.0 ± 1.2	26.6 ± 0.6	28.6 ± 1.2	21.3 ± 3.2	22.0 ± 1.7	20.4 ± 1.8	27.9 ± 2.7	17.1 ± 2.2	25.9 ± 1.2
n	8	6	6	3	3	6	8	8	3	3
P	–	0.28	0.08	**0.049**	0.67	> 0.99	0.7	0.07	0.17	0.28
Hill Slopes	2.5 ± 0.2	3.0 ± 0.2	2.4 ± 0.1	3.0 ± 0.2	3.3 ± 0.4	2.8 ± 0.2	4.0 ± 0.3	2.9 ± 0.1	5.2 ± 0.7	3.5 ± 0.5
LUM IC_{50}	1.03 ± 0.07	1.30 ± 0.14	1.07 ± 0.04	1.09 ± 0.07	1.37 ± 0.21	1.32 ± 0.10	2.13 ± 0.13	1.08 ± 0.10	2.08 ± 0.07	1.07 ± 0.03
n	6	5	5	2	2	5	6	6	3	2
P	–	0.13	0.65	0.64	0.14	0.052	**0.002**	0.91	**0.024**	0.86
LUM IC_{90}	4.63 ± 0.37	4.94 ± 0.41	4.42 ± 0.21	4.60 ± 0.05	4.98 ± 0.56	5.40 ± 0.58	7.56 ± 0.47	5.73 ± 0.81	8.33 ± 0.31	6.52 ± 0.57
n	6	5	5	2	2	5	6	6	3	2
P	–	0.77	0.87	0.64	0.43	0.42	**0.004**	0.42	**0.024**	0.14
Hill Slopes	1.7 ± 0.1	1.6 ± 0.1	1.9 ± 0.1	1.3 ± 0.2	2.2 ± 0.2	1.9 ± 0.1	2.0 ± 0.2	1.7 ± 0.1	1.8 ± 0.1	1.6 ± 0.2
AS IC_{50}	0.67 ± 0.08	0.84 ± 0.12	0.52 ± 0.07	0.86 ± 0.05	0.83 ± 0.16	0.73 ± 0.08	1.17 ± 0.13	0.73 ± 0.11	0.90 ± 0.01	1.83 ± 0.21
n	7	5	5	7	2	5	7	7	3	6
P	–	0.29	0.2	0.18	0.39	0.56	**0.007**	0.56	0.18	**0.0012**
AS IC_{90}	2.31 ± 0.36	2.33 ± 0.29	1.94 ± 0.08	3.37 ± 0.33	2.24 ± 0.03	2.31 ± 0.11	3.57 ± 0.35	2.53 ± 0.45	2.83 ± 0.29	4.03 ± 0.41
n	7	5	5	7	2	5	7	7	3	6
P	–	0.55	0.5	0.053	0.5	0.36	**0.018**	0.31	0.18	**0.022**
Hill Slopes	1.9 ± 0.2	2.6 ± 0.3	1.7 ± 0.4	2.0 ± 0.2	2.6 ± 0.6	2.2 ± 0.3	2.7 ± 0.2	2.1 ± 0.4	2.4 ± 0.2	4.2 ± 0.4

S2 Table (page 3 of 3).

	$GC03^{Cam734}$	$GC03^{Cam734}$ back-mutants					$GC03^{GC03}$	$GC03^{Dd2}$	GC03	Dd2
		D75N	F144A	I148L	T194I	S333T				
PND IC_{50}	5.0 ± 0.3	6.2 ± 0.3	5.9 ± 0.3	5.0 ± 0.4	5.6 ± 0.8	5.9 ± 0.3	6.5 ± 0.2	5.3 ± 0.5	5.8 ± 0.4	5.7 ± 0.7
n	8	4	4	3	3	4	6	6	5	3
P	–	0.073	0.15	0.78	0.5	0.15	**0.008**	0.41	0.17	0.19
PND IC_{90}	7.9 ± 0.4	8.7 ± 0.1	8.7 ± 0.1	8.4 ± 0.6	8.8 ± 0.3	8.7 ± 0.1	9.7 ± 0.6	9.1 ± 0.6	8.7 ± 0.1	8.7 ± 0.1
n	8	4	4	3	3	4	6	6	5	3
P	–	0.28	0.28	0.5	0.28	0.48	**0.011**	0.34	0.18	0.19
Hill Slopes	5.4 ± 0.8	7.8 ± 1.4	6.4 ± 0.9	4.9 ± 0.2	4.4 ± 0.9	6.5 ± 0.9	7.4 ± 1.1	4.9 ± 0.7	7.1 ± 1.0	7.3 ± 1.6

IC_{50} and IC_{90} values (nM) indicate the mean ± SEM, as determined in 2 to 12 independent assays performed in duplicate. CQ + VP assays were performed with 0.8 µM VP. CQ, chloroquine; VP, verapamil; md-CQ, monodesethyl-chloroquine; md-AQ, monodesethyl-amodiaquine; QN, quinine; PPQ, piperaquine; LUM, lumefantrine; AS, artesunate; PND, pyronaridine; n, number of assays. *P* values were determined in a non-parametric Mann-Whitney *U* test versus the parasite line $GC03^{Cam734}$. *P* values <0.05 are indicated in bold and shaded in gray. Hill slopes were calculated from the dose-response data using GraphPad Prism 6 software.
