# Supplementary material for: Evolution of Fitness Cost-Neutral Mutant PfCRT Conferring P. falciparum 4-Aminoquinoline Drug Resistance Is Accompanied by Altered Parasite Metabolism and Digestive Vacuole Physiology
Source: PLoS Pathog. 2016 Nov 10;12(11):e1005976. doi: 10.1371/journal.ppat.1005976 (PMC5104409; doi:10.1371/journal.ppat.1005976)
Supplement: S3 Table — Reversibility of chloroquine (CQ) resistance by 0.8 μM verapamil (VP) is indicated as the CQ response modification index (RMI), equivalent to (IC50 for CQ+VP) ÷ (IC50 for CQ only). Shown are mean RMI ± SEM values, as determined in 5 to 12 independent assays. n, number of assays. P values were determined in a non-parametric Mann-Whitney U test versus the parasite line GC03Cam734. P values <0.05 are indicated in bold and shaded in gray. (PDF) [file ppat.1005976.s010.pdf]

S3 Table. Verapamil-mediated CQ resistance reversibility of *pfcrt*-modified and reference lines.

Line			Line			Line		
$GC03^{Cam734}$	RMI	0.54 ± 0.04	$GC03^{Cam734\ T194I}$	RMI	0.59 ± 0.03	GC03	RMI	1.2 ± 0.21
	n	12		*n*	5		*n*	5
	P	–		*P*	0.63		*P*	**0.025**
$GC03^{Cam734\ D75N}$	RMI	0.92 ± 0.15	$GC03^{Cam734\ S333T}$	RMI	0.57 ± 0.11	Dd2	RMI	0.31 ± 0.04
	n	6		*n*	6		*n*	5
	P	**0.02**		*P*	0.84		*P*	**0.0048**
$GC03^{Cam734\ F144A}$	RMI	0.90 ± 0.17	$GC03^{GC03}$	RMI	1.3 ± 0.15			
	n	6		*n*	10			
	P	0.13		*P*	**< 0.0001**			
$GC03^{Cam734\ I148L}$	RMI	0.93 ± 0.07	$GC03^{Dd2}$	RMI	0.16 ± 0.03			
	n	5		*n*	10			
	P	**0.0002**		*P*	**< 0.0001**			

Reversibility of chloroquine (CQ) resistance by 0.8 µM verapamil (VP) is indicated as the CQ response modification index (RMI), equivalent to (IC_{50} for CQ+VP) ÷ (IC_{50} for CQ only). Shown are mean RMI ± SEM values, as determined in 5 to 12 independent assays. *n*, number of assays. *P* values were determined in a non-parametric Mann-Whitney *U* test versus the parasite line $GC03^{Cam734}$. *P* values <0.05 are indicated in **bold** and shaded in gray.
