# Supplementary material for: Evolution of Fitness Cost-Neutral Mutant PfCRT Conferring P. falciparum 4-Aminoquinoline Drug Resistance Is Accompanied by Altered Parasite Metabolism and Digestive Vacuole Physiology
Source: PLoS Pathog. 2016 Nov 10;12(11):e1005976. doi: 10.1371/journal.ppat.1005976 (PMC5104409; doi:10.1371/journal.ppat.1005976)
Supplement: S4 Table — Parasite in vitro growth was evaluated in the absence or presence of a sub-lethal dose of CQ (7.5 nM; ~0.5× CQ IC50 of the CQ-sensitive reference line GC03GC03) and normalized against GC03Cam734 in the absence of drug pressure (6 total replicates per condition). As detailed in Materials and Methods, the per-generation selection coefficient (indicated above as s ± SEM) was derived from the relative fitness index (ω') as per the relationship s = ω'– 1, such that s<0 and s>0 respectively indicate growth inferior or superior to the GC03Cam734 parasite line, which encodes the full-length Cam734 pfcrt allele. CQ, chloroquine; P 1, P value for inter-strain comparisons, determined versus the parasite line GC03Cam734 using two-way ANOVA with Sidak’s post-hoc test; P 2, P value for intra-strain comparisons, determined for a given parasite strain in the absence versus presence of 7.5 nM CQ using two-way ANOVA with Sidak’s post-hoc test. P values <0.05 are indicated in bold and shaded in gray. (PDF) [file ppat.1005976.s011.pdf]

S4 Table. *In vitro* growth selection coefficients of *pfcrt*-modified and reference parasite lines.

Line		No drug	7.5 nM CQ	Line		No drug	7.5 nM CQ
	s	0.00 ± 0.008	0.18 ± 0.026		*s*	-0.17 ± 0.008	-0.19 ± 0.009
GC03^{Cam734}	P_1	–	–	GC03$^{Cam734\ S333T}$	P_1	**< 0.0001**	**< 0.0001**
	P_2	**< 0.0001**			P_2	0.69	
	s	-0.11 ± 0.009	-0.11 ± 0.011		*s*	0.06 ± 0.007	0.03 ± 0.008
GC03$^{Cam734\ D75N}$	P_1	**< 0.0001**	**< 0.0001**	GC03^{GC03}	P_1	**0.002**	**< 0.0001**
	P_2	> 0.99			P_2	0.71	
	s	-0.07 ± 0.009	-0.14 ± 0.010		*s*	-0.25 ± 0.006	-0.25 ± 0.007
GC03$^{Cam734\ F144A}$	P_1	**< 0.0001**	**< 0.0001**	GC03^{Dd2}	P_1	**< 0.0001**	**< 0.0001**
	P_2	**< 0.0001**			P_2	> 0.99	
	s	-0.26 ± 0.006	-0.30 ± 0.007		*s*	-0.05 ± 0.009	-0.06 ± 0.009
GC03$^{Cam734\ I148L}$	P_1	**< 0.0001**	**< 0.0001**	GC03	P_1	**0.0047**	**< 0.0001**
	P_2	0.15			P_2	0.99	
	s	-0.14 ± 0.009	-0.15 ± 0.009				
GC03$^{Cam734\ T194I}$	P_1	**< 0.0001**	**< 0.0001**				
	P_2	0.99					

Parasite *in vitro* growth was evaluated in the absence or presence of a sub-lethal dose of CQ (7.5 nM; ~0.5×CQ IC_{50} of the CQ-sensitive reference line GC03^{GC03}) and normalized against GC03^{Cam734} in the absence of drug pressure (6 replicates per condition). As detailed in **Materials and Methods**, the per-generation selection coefficient (indicated above as *s* ± SEM) was derived from the relative fitness index (ω') as per the relationship $s = \omega' - 1$, such that $s<0$ and $s>0$ respectively indicate growth inferior or superior to the GC03^{Cam734} parasite line, which encodes the full-length Cam734 *pfcrt* allele. CQ, chloroquine; P_1, *P* value for inter-strain comparisons, determined versus the parasite line GC03^{Cam734} using two-way ANOVA with Sidak's post-hoc test; P_2, *P* value for intra-strain comparisons, determined for a given parasite strain in the absence versus presence of 7.5 nM CQ using two-way ANOVA with Sidak's post-hoc test. *P* values <0.05 are indicated in **bold** and shaded in gray.
