# Supplementary material for: Evolution of Fitness Cost-Neutral Mutant PfCRT Conferring P. falciparum 4-Aminoquinoline Drug Resistance Is Accompanied by Altered Parasite Metabolism and Digestive Vacuole Physiology
Source: PLoS Pathog. 2016 Nov 10;12(11):e1005976. doi: 10.1371/journal.ppat.1005976 (PMC5104409; doi:10.1371/journal.ppat.1005976)
Supplement: S5 Table — Mass spectrometric signal intensities for metabolites derived from trophozoite-stage isogenic (GC03) parasites encoding either Dd2 or Cam734 pfcrt, as detailed in Materials and Methods. Metabolites were harvested on three independent occasions, yielding 4 and 6 individual replicates (r) for the GC03Dd2 and GC03Cam734 strains, respectively. (PDF) [file ppat.1005976.s012.pdf]

S5 Table. Metabolite mass spectrometry signal intensities (page 1 of 2).

		GC03^{Dd2}				GC03^{Cam734}					
**Class**	**Compound**	***r1***	***r2***	***r3***	***r4***	***r1***	***r2***	***r3***	***r4***	***r5***	***r6***
**Glycolysis**	2,3-bisphosphoglycerate (2,3-BPG)	1,818,426	2,041,584	1,754,390	2,125,341	2,622,063	2,608,565	2,551,841	2,308,076	3,383,943	3,704,857
	3-phosphoglycerate (3-PG)	167,233	327,019	175,006	255,962	261,910	274,101	333,140	331,468	495,002	562,054
	D-glyceraldehdye-3-phosphate (PGAL)	9,980	13,791	10,403	12,606	12,380	11,891	12,802	11,976	11,832	15,463
	Dihydroxy-acetone-phosphate (DHAP)	3,300	4,871	2,884	3,986	3,957	4,623	4,197	5,298	7,424	7,602
	Fructose-1,6-bisphosphate (F-1,6-BP)	645,797	680,613	540,089	762,884	852,362	829,851	847,985	927,166	1,467,163	1,491,719
	Glucose-6-phosphate (G-6-P)	1,476,761	2,011,221	1,423,394	1,727,964	1,871,048	1,938,490	1,948,432	2,065,075	1,904,503	2,137,552
	Phosphoenolpyruvate (PEP)	2,783,847	3,133,988	2,649,281	3,131,835	4,156,352	3,897,813	3,720,144	3,550,549	4,638,344	5,957,708
	sn-Glycerol-3-phosphate (sn-Glycerol-3-P)	676,694	898,562	544,931	809,045	908,133	780,598	750,256	799,847	803,109	855,003
**TCA-linked**	4-aminobutyrate (GABA)	398,251	430,053	386,426	421,349	491,355	472,599	526,773	500,132	453,322	489,160
	α-ketoglutarate (α-KG)	17,151	20,468	18,678	23,428	26,113	27,665	22,811	21,976	30,071	28,874
	Citrate	63,577	90,109	68,633	73,222	87,721	96,898	93,136	100,150	100,387	91,874
	Fumarate	42,264	98,518	30,822	58,270	56,402	49,428	59,054	62,444	57,168	80,374
	Glutamate	730,007	824,672	694,324	686,559	904,280	826,855	994,747	945,465	881,883	1,030,638
	Malate	58,699	91,489	48,797	76,840	82,591	81,084	69,052	81,288	72,914	89,851
	Succinate	67,614	103,454	67,778	92,821	79,939	79,475	106,142	96,435	106,585	108,453
**Pentose phosphate pathway**	6-phospho-D-gluconate (6-P-D-gluconate)	145,807	280,379	147,462	230,122	210,341	212,360	223,480	254,293	231,634	298,599
	D-erythrose-4-phosphate (D-erythrose-4-P)	47,768	73,022	47,980	64,139	61,373	63,560	61,990	59,953	55,833	72,027
	D-gluconate	264,704	306,988	189,628	248,342	244,144	206,670	213,861	240,275	254,269	264,465
	D-sedoheptulose-7-phosphate (D-S-7-P)	62,289	95,386	49,191	64,687	97,028	84,734	85,422	84,832	58,341	72,712
	Octulose-1-phosphate (Octulose-1-P)	10,234	13,432	7,491	9,225	10,648	9,541	11,079	10,292	11,871	12,431
	Octulose-bisphosphate (Octulose-BP)	33,390	48,096	30,754	37,211	40,706	37,982	43,659	42,478	45,093	47,776
	Ribose-5-phosphate (Rib-5-P)	27,387	47,890	26,706	36,946	40,693	38,611	46,681	43,321	34,860	40,094
	Sedoheptulose bisphosphate (S-BP)	66,229	91,582	49,798	74,506	82,768	83,296	82,794	89,561	97,384	120,354
	Xylulose-5-phosphate (Xyl-5-P)	22,021	40,591	20,567	27,345	27,116	26,078	32,473	30,861	29,632	27,051
**Nucleotide metabolism/ energy**	N-Carbamoyl-L-aspartate (N-carb-L-Asp)	57,278	53,724	29,573	31,440	85,509	78,013	36,554	40,906	40,959	48,331
	ATP	12,200,000	15,900,000	14,100,000	15,600,000	19,700,000	18,200,000	21,600,000	20,800,000	18,400,000	20,100,000
	ADP	1,677,320	2,498,387	1,903,922	2,006,714	1,810,411	1,566,066	1,849,525	1,951,828	2,042,172	1,792,070
	AMP	172,159	321,038	221,593	206,935	168,104	143,568	181,286	190,480	221,660	194,309
	dATP	143,726	331,088	232,224	250,158	347,101	321,788	369,775	373,811	335,195	383,815
	dAMP	1,952	5,407	3,708	2,508	2,237	1,981	2,319	2,347	2,923	2,201
	CTP	108,940	183,300	135,428	133,305	213,026	196,137	195,420	201,028	192,681	174,255
	CDP	40,596	76,673	48,328	45,580	43,803	37,451	43,989	45,390	45,504	38,786
	CMP	43,704	67,030	39,707	47,094	29,045	26,979	35,138	32,395	30,291	27,579
	dCTP	17,539	34,031	25,274	24,988	25,337	22,768	30,655	32,513	24,340	24,108
	dTTP	443,330	1,010,352	709,077	856,277	1,092,340	989,572	1,318,475	1,202,133	973,540	1,151,239
	dTDP	32,294	60,650	37,823	41,959	30,988	24,554	33,785	34,952	34,679	30,602
	GTP	115,657	240,040	192,244	187,484	322,574	284,909	344,057	324,339	319,786	307,135
	GDP	74,386	110,184	94,753	84,101	79,994	69,796	87,570	79,771	81,611	60,698
	GMP	236,587	421,961	286,234	352,737	145,391	147,434	281,107	286,117	188,463	182,225
	UTP	1,158,378	2,394,405	1,767,786	1,882,838	2,826,131	2,538,610	2,828,001	2,770,722	3,044,939	3,211,305
	UDP	261,516	441,973	324,190	335,686	246,599	204,514	257,486	254,860	316,185	278,371
	UMP	240,654	392,608	273,955	339,769	124,319	135,632	193,225	198,434	183,300	186,591
	IMP	668,862	933,780	566,465	756,838	790,513	831,408	818,740	858,756	837,974	1,036,262
	Inosine (I)	15,953	35,175	17,214	23,596	22,231	22,950	22,192	23,283	23,261	29,979
	Thymidine (dT)	960	5,269	2,091	2,544	2,717	4,740	2,556	2,854	3,113	4,199

S5 Table. Metabolite mass spectrometry signal intensities (page 2 of 2).

		GC03Dd2				GC03Cam734					
**Class**	**Compound**	***r1***	***r2***	***r3***	***r4***	***r1***	***r2***	***r3***	***r4***	***r5***	***r6***
**Nucleotide conjugate**	ADP-D-glucose	85,070	107,651	80,044	98,144	100,150	97,310	100,485	105,685	105,492	123,345
	CDP-choline	13,307	15,414	11,079	14,286	12,386	12,226	11,545	11,600	11,360	14,397
	CDP-ethanolamine (CDP-ETA)	742,782	781,198	627,327	806,594	629,475	640,599	598,245	616,761	629,968	834,158
	S-Adenosyl-L-homocysteine (SAH)	95,401	235,866	112,320	166,585	123,896	108,728	150,741	156,159	107,269	163,194
	UDP-D-glucose	11,800,000	5,441,816	5,202,778	7,165,272	5,908,932	5,172,436	4,319,636	4,659,530	4,867,554	5,532,844
	UDP-D-glucuronate	203,847	300,276	201,951	245,545	344,429	301,591	383,076	346,847	346,890	378,313
	UDP-N-acetyl-glucosamine (UDP-NAG)	3,573,439	3,688,092	2,430,914	3,401,627	3,035,240	2,784,650	2,957,299	3,261,140	2,701,282	3,374,203
**Redox**	Glutathione (GSH)	24,383	54,251	19,424	50,727	11,490	9,869	19,513	24,489	24,578	37,344
	Glutathione disulfide (GSSG)	9,604,691	12,500,000	7,124,940	11,500,000	9,262,997	9,202,598	10,700,000	10,900,000	10,400,000	12,300,000
	NAD+	1,820,134	2,726,520	1,645,891	2,258,642	1,987,036	1,720,146	2,000,814	2,095,764	1,952,684	2,694,296
	NADH	8,079	8,094	8,746	11,113	8,233	6,727	7,557	9,856	12,086	13,722
	NADP+	354,352	517,714	323,339	363,667	420,009	429,742	463,162	463,212	429,282	459,912
**Amino acid**	Phenylalanine (Phe)	391,301	510,463	407,452	415,334	492,426	490,525	533,622	480,780	489,014	490,636
	Tryptophan (Trp)	421,922	514,285	444,772	452,192	519,217	555,928	484,672	529,143	552,688	513,104
**N-acetylated amino acid**	N-Acetyl-glucosamine-1,6-bisphosphate (NAG-1,6-BP)	16,540	28,008	16,426	24,512	19,231	17,075	18,847	23,319	25,032	28,138
	N-Acetyl-glutamate (NAc-Glu)	8,994	15,352	6,170	12,155	12,733	15,724	11,388	14,677	14,109	16,849
	N-Acetyl-glutamine (NAc-Gln)	21,829	33,938	23,217	32,363	33,753	33,154	38,308	42,070	42,315	49,967
	N-Acetyl-L-alanine (NAc-L-Ala)	3,029	4,010	2,764	4,596	4,153	3,768	3,907	4,163	2,880	4,051
**Peptide**	DLH	2,278,636	4,337,030	2,603,910	3,338,881	1,766,444	1,687,534	1,865,405	1,988,353	1,380,088	1,642,514
	DLS	1,352,593	2,257,624	1,615,609	2,270,828	1,882,258	1,806,078	1,701,533	1,859,163	1,811,037	2,091,223
	HVDD	2,314,579	3,475,401	2,301,480	3,209,824	2,614,628	2,566,558	2,086,442	2,242,085	2,124,340	2,399,405
	LD	262,781	432,318	157,071	230,188	373,873	374,007	337,524	354,245	243,135	334,855
	PD	2,603,180	3,210,624	2,524,118	3,485,913	2,582,608	2,711,633	2,660,678	2,852,841	2,867,926	3,697,181
	PE	3,301,973	4,022,815	3,106,118	4,016,915	3,948,352	3,436,766	3,796,633	3,862,571	3,272,647	4,153,873
	PEE	1,865,340	2,744,282	1,797,025	2,537,866	2,422,479	2,356,507	2,479,936	2,760,543	1,977,875	2,504,034
	PEEK	189,185	221,324	188,229	237,220	264,308	256,996	239,572	255,284	234,097	311,831
	PVNF	1,360,635	2,338,811	1,487,999	1,884,430	1,703,102	1,715,251	1,728,604	1,754,043	1,310,169	1,421,994
	SD	171,744	239,817	160,016	219,871	196,145	194,951	204,022	218,614	192,478	229,862
	VD	45,135	59,271	38,739	54,490	57,423	52,634	54,671	50,986	47,372	63,206
**Cofactor**	Biotin	25,848	39,272	30,570	25,713	39,626	45,999	38,780	39,643	37,957	36,059
	Folate	127,338	123,295	149,209	129,109	160,356	161,083	183,411	194,285	190,937	169,831
	Pantothenate	86,643	165,431	95,293	102,700	165,705	157,496	131,378	146,210	124,089	119,590

Mass spectrometric signal intensities for metabolites derived from trophozoite-stage isogenic (GC03) parasites encoding either Dd2 or Cam734 *pfcrt,* as detailed in **Materials and Methods.** Metabolites were harvested on three independent occasions, yielding 4 and 6 individual replicates (r) for the GC03Dd2 and GC03Cam734 strains, respectively.
