# Supplementary material for: Evolution of Fitness Cost-Neutral Mutant PfCRT Conferring P. falciparum 4-Aminoquinoline Drug Resistance Is Accompanied by Altered Parasite Metabolism and Digestive Vacuole Physiology
Source: PLoS Pathog. 2016 Nov 10;12(11):e1005976. doi: 10.1371/journal.ppat.1005976 (PMC5104409; doi:10.1371/journal.ppat.1005976)
Supplement: S6 Table — Metabolite classes and individual metabolite z-scores (see Materials and Methods for z-score derivation). P values were determined using one-way ANOVA. Corresponding metabolite signal intensities are reported in S5 Table. Metabolites were harvested on three independent occasions, yielding 4 and 6 individual replicates (r) for the GC03Dd2 and GC03Cam734 strains, respectively. P values <0.05 are shaded in gray. (PDF) [file ppat.1005976.s013.pdf]

S6 Table. Metabolite *z*-scores and *P* values (page 1 of 2).

		GC03^{Dd2}				GC03^{Cam734}						
**Compound class or compound**	**Class**	***r1***	***r2***	***r3***	***r4***	***r1***	***r2***	***r3***	***r4***	***r5***	***r6***	***P* value**
**Compound classes**												
ATP/ADP		0.116	-1.403	0.336	0.951	6.141	7.376	7.472	5.765	3.015	6.699	0.0002
ATP/AMP		0.532	-1.355	-0.108	0.932	4.629	5.476	4.802	3.922	1.606	3.413	0.0011
Glycolysis		-0.593	0.987	-1.090	0.696	2.616	2.263	2.080	1.905	4.706	6.794	0.0133
Pentose phosphate pathway (PPP)		-0.438	1.300	-1.042	0.179	0.301	0.016	0.228	0.526	0.324	1.071	0.3740
Tricarboxylic acid (TCA) cycle		-0.458	1.421	-0.873	-0.091	1.887	1.256	2.844	2.418	1.713	3.162	0.0035
Nucleotide biosynthesis		0.275	1.276	-0.993	-0.557	2.092	1.854	-0.371	-0.083	-0.069	0.692	0.3360
Redox		-0.316	1.056	-1.239	0.499	-0.358	-0.468	0.158	0.262	0.030	0.953	0.8439
Cofactors		-0.926	1.389	-0.001	-0.462	2.378	2.349	2.060	2.758	2.045	1.323	0.0017
Nucleoside triphosphates (nTPs)		-1.329	0.952	-0.169	0.546	2.882	2.064	3.729	3.342	2.391	3.207	0.0004
Nucleoside diphosphates (nDPs)		-1.003	1.378	-0.299	-0.075	-0.722	-1.394	-0.593	-0.386	-0.046	-0.746	0.1939
Nucleoside monophosphates (nMPs)		-0.791	1.354	-0.719	0.155	-1.080	-1.004	-0.382	-0.225	-0.514	-0.057	0.2602
Deoxynucleoside triphosphates (dnTPs)		-1.282	1.100	-0.164	0.346	1.376	0.972	2.161	1.820	0.969	1.668	0.0123
Deoxynucleoside diphosphates (dnDPs)		-0.885	1.420	-0.436	-0.099	-0.991	-1.514	-0.764	-0.669	-0.691	-1.023	0.0586
Deoxynucleoside monophosphates (dnMPs)		-0.943	1.317	0.205	-0.579	-0.756	-0.924	-0.703	-0.684	-0.308	-0.780	0.1290
Nucleotide conjugates		1.377	-0.401	-0.971	-0.004	-0.526	-0.836	-1.015	-0.824	-0.940	-0.446	0.1006
N-Acetyl (NAc)-Amino Acids		-0.794	1.082	-0.904	0.616	0.388	0.379	0.545	1.260	1.266	2.157	0.0974
Aromatic amino acids		-0.819	1.454	-0.400	-0.235	1.313	1.687	1.385	1.295	1.636	1.228	0.0081
Peptides		-0.879	1.089	-0.819	0.609	-0.344	-0.513	-0.514	-0.243	-0.953	-0.075	0.3296
**Individual compounds**												
2,3-bisphosphoglycerate (2,3-BPG)	Glycolysis	-0.659	0.603	-1.021	1.077	3.886	3.810	3.489	2.110	8.195	10.010	0.0123
3-phosphoglycerate (3-PG)	Glycolysis	-0.850	1.270	-0.747	0.327	0.406	0.568	1.351	1.329	3.498	4.388	0.0708
D-glyceraldehdye-3-phosphate (PGAL)	Glycolysis	-0.947	1.158	-0.714	0.503	0.378	0.108	0.611	0.155	0.075	2.081	0.3372
Dihydroxy-acetone-phosphate (DHAP)	Glycolysis	-0.530	1.278	-1.009	0.260	0.227	0.993	0.502	1.771	4.219	4.424	0.0844
Fructose-1,6-bisphosphate (F-1,6-BP)	Glycolysis	-0.125	0.252	-1.270	1.143	2.113	1.869	2.065	2.923	8.773	9.039	0.0389
Glucose-6-phosphate (G-6-P)	Glycolysis	-0.680	1.305	-0.878	0.253	0.784	1.035	1.072	1.505	0.909	1.774	0.0279
Phosphoenolpyruvate (PEP)	Glycolysis	-0.571	0.849	-1.117	0.840	4.995	3.946	3.226	2.538	6.950	12.300	0.0168
sn-Glycerol-3-phosphate (sn-Glycerol-3-P)	Glycolysis	-0.360	1.075	-1.212	0.496	1.137	0.312	0.116	0.437	0.458	0.793	0.2500
4-aminobutyrate (GABA)	TCA-associated	-0.534	1.043	-1.120	0.611	4.081	3.152	5.837	4.517	2.196	3.973	0.0007
α-ketoglutarate (α-KG)	TCA-associated	-1.031	0.199	-0.465	1.297	2.292	2.868	1.068	0.758	3.760	3.316	0.0128
Citrate	TCA-associated	-0.896	1.409	-0.456	-0.058	1.202	1.999	1.672	2.282	2.302	1.563	0.0037
Fumarate	TCA-associated	-0.514	1.387	-0.900	0.027	-0.036	-0.272	0.054	0.168	-0.010	0.774	0.8015
Glutamate	TCA-associated	-0.061	1.432	-0.624	-0.746	2.687	1.466	4.114	3.337	2.334	4.680	0.0026
Malate	TCA-associated	-0.540	1.187	-1.062	0.415	0.718	0.639	0.005	0.649	0.208	1.101	0.2466
Succinate	TCA-associated	-0.845	1.134	-0.836	0.547	-0.164	-0.190	1.283	0.747	1.307	1.411	0.2177
6-phospho-D-gluconate (6-P-D-gluconate)	Pentose phosphate pathway	-0.836	1.204	-0.811	0.442	0.142	0.173	0.342	0.809	0.465	1.480	0.2634
D-erythrose-4-phosphate (D-erythrose-4-P)	Pentose phosphate pathway	-0.837	1.184	-0.820	0.473	0.252	0.427	0.301	0.138	-0.192	1.105	0.4755
D-gluconate	Pentose phosphate pathway	0.253	1.123	-1.292	-0.084	-0.170	-0.941	-0.793	-0.250	0.038	0.248	0.5188
D-sedoheptulose-7-phosphate (D-S-7-P)	Pentose phosphate pathway	-0.286	1.406	-0.956	-0.164	1.490	0.861	0.897	0.866	-0.488	0.247	0.2548
Octulose-1-phosphate (Octulose-1-P)	Pentose phosphate pathway	0.055	1.337	-1.043	-0.349	0.221	-0.222	0.394	0.079	0.711	0.936	0.4555
Octulose-bisphosphate (Octulose-BP)	Pentose phosphate pathway	-0.521	1.407	-0.866	-0.020	0.438	0.081	0.825	0.670	1.013	1.365	0.1475
Ribose-5-phosphate (Rib-5-P)	Pentose phosphate pathway	-0.739	1.324	-0.807	0.223	0.600	0.390	1.202	0.864	0.013	0.539	0.2147
Sedoheptulose bisphosphate (S-BP)	Pentose phosphate pathway	-0.247	1.211	-1.192	0.229	0.704	0.734	0.705	1.094	1.544	2.865	0.0611
Xylulose-5-phosphate (Xyl-5-P)	Pentose phosphate pathway	-0.615	1.421	-0.775	-0.031	-0.057	-0.170	0.531	0.354	0.219	-0.064	0.7547
N-Carbamoyl-L-aspartate (N-carb-L-Asp)	Energy/nucleotide metabolism	0.983	0.738	-0.925	-0.796	2.927	2.411	-0.444	-0.144	-0.141	0.367	0.3538
ATP	Energy/nucleotide metabolism	-1.328	0.856	-0.207	0.679	3.099	2.214	4.221	3.748	2.332	3.335	0.0005
ADP	Energy/nucleotide metabolism	-0.994	1.377	-0.340	-0.043	-0.610	-1.315	-0.497	-0.201	0.059	-0.663	0.2779
AMP	Energy/nucleotide metabolism	-0.912	1.419	-0.138	-0.368	-0.976	-1.360	-0.770	-0.626	-0.137	-0.566	0.1374
dATP	Energy/nucleotide metabolism	-1.243	1.194	-0.092	0.141	1.402	1.073	1.697	1.750	1.247	1.880	0.0077
dAMP	Energy/nucleotide metabolism	-0.943	1.317	0.205	-0.579	-0.756	-0.924	-0.703	-0.684	-0.308	-0.780	0.1290
CTP	Energy/nucleotide metabolism	-1.006	1.384	-0.155	-0.223	2.339	1.796	1.773	1.953	1.685	1.093	0.0041
CDP	Energy/nucleotide metabolism	-0.751	1.471	-0.275	-0.444	-0.554	-0.945	-0.542	-0.456	-0.449	-0.863	0.1603
CMP	Energy/nucleotide metabolism	-0.468	1.453	-0.797	-0.189	-1.675	-1.845	-1.173	-1.399	-1.572	-1.795	0.0053
dCTP	Energy/nucleotide metabolism	-1.174	1.271	-0.027	-0.070	-0.018	-0.399	0.771	1.046	-0.166	-0.200	0.7377
dTTP	Energy/nucleotide metabolism	-1.291	1.059	-0.189	0.421	1.399	0.973	2.336	1.854	0.907	1.643	0.0137
dTDP	Energy/nucleotide metabolism	-0.885	1.420	-0.436	-0.099	-0.991	-1.514	-0.764	-0.669	-0.691	-1.023	0.0586
GTP	Energy/nucleotide metabolism	-1.330	1.095	0.164	0.071	2.705	1.970	3.124	2.739	2.650	2.404	0.0004
GDP	Energy/nucleotide metabolism	-1.074	1.260	0.254	-0.440	-0.708	-1.373	-0.214	-0.723	-0.603	-1.966	0.1049
GMP	Energy/nucleotide metabolism	-1.089	1.211	-0.473	0.352	-2.221	-2.195	-0.537	-0.475	-1.686	-1.764	0.0302
UTP	Energy/nucleotide metabolism	-1.266	1.169	-0.065	0.162	2.020	1.453	2.024	1.911	2.451	2.779	0.0018
UDP	Energy/nucleotide metabolism	-1.059	1.350	-0.222	-0.069	-1.258	-1.820	-1.113	-1.148	-0.329	-0.834	0.0493
UMP	Energy/nucleotide metabolism	-1.048	1.192	-0.557	0.413	-2.763	-2.596	-1.747	-1.670	-1.893	-1.845	0.0020
IMP	Energy/nucleotide metabolism	-0.402	1.299	-1.060	0.163	0.379	0.642	0.560	0.817	0.684	1.958	0.1246

S6 Table. Metabolite *z*-scores and *P* values (page 2 of 2).

Individual compounds (continued)	Class (continued)											
Inosine (I)	Energy/nucleotide metabolism	-0.800	1.387	-0.657	0.070	-0.086	-0.004	-0.090	0.034	0.031	0.796	0.7990
Thymidine (dT)	Energy/nucleotide metabolism	-0.961	1.397	-0.342	-0.094	0.001	1.107	-0.087	0.075	0.217	0.811	0.4704
ADP-D-glucose	Nucleotide conjugate	-0.611	1.190	-1.012	0.432	0.592	0.366	0.619	1.034	1.018	2.442	0.1028
CDP-choline	Nucleotide conjugate	-0.116	1.028	-1.326	0.415	-0.616	-0.703	-1.073	-1.044	-1.174	0.476	0.2084
CDP-ethanolamine (CDP-ETA)	Nucleotide conjugate	0.042	0.527	-1.415	0.847	-1.388	-1.248	-1.782	-1.549	-1.382	1.195	0.1743
S-Adenosyl-L-homocysteine (SAH)	Nucleotide conjugate	-0.903	1.316	-0.635	0.222	-0.453	-0.692	-0.028	0.057	-0.715	0.168	0.5489
UDP-D-glucose	Nucleotide conjugate	1.437	-0.641	-0.719	-0.078	-0.488	-0.729	-1.008	-0.897	-0.829	-0.611	0.0988
UDP-D-glucuronate	Nucleotide conjugate	-0.737	1.350	-0.778	0.165	2.306	1.379	3.143	2.358	2.359	3.040	0.0014
UDP-N-acetyl-glucosamine (UDP-NAG)	Nucleotide conjugate	0.523	0.722	-1.468	0.223	-0.415	-0.852	-0.551	-0.022	-0.997	0.175	0.3623
Glutathione (GSH)	Redox metabolism	-0.719	0.956	-0.997	0.759	-1.442	-1.532	-0.992	-0.713	-0.708	0.008	0.1044
Glutathione disulfide (GSSG)	Redox metabolism	-0.244	0.980	-1.292	0.557	-0.389	-0.414	0.219	0.303	0.092	0.895	0.8073
NAD+	Redox metabolism	-0.605	1.269	-0.965	0.302	-0.260	-0.812	-0.232	-0.035	-0.331	1.202	0.8858
NADH	Redox metabolism	-0.646	-0.636	-0.182	1.465	-0.539	-1.587	-1.010	0.590	2.142	3.280	0.6599
NADP+	Redox metabolism	-0.407	1.470	-0.763	-0.300	0.348	0.459	0.843	0.844	0.454	0.806	0.1675
Phenylalanine (Phe)	Amino acid	-0.740	1.474	-0.440	-0.294	1.139	1.103	1.904	0.922	1.075	1.105	0.0237
Tryptophan (Trp)	Amino acid	-0.921	1.418	-0.342	-0.154	1.543	2.473	0.668	1.794	2.390	1.388	0.0115
N-Acetyl-glucosamine-1,6-bisphosphate (NAG-1,6-BP)	Amino acid conjugate	-0.830	1.140	-0.849	0.539	-0.368	-0.738	-0.434	0.334	0.629	1.162	0.8618
N-Acetyl-glutamate (NAc-Glu)	Amino acid conjugate	-0.422	1.181	-1.134	0.375	0.521	1.275	0.182	1.011	0.868	1.559	0.0913
N-Acetyl-glutamine (NAc-Gln)	Amino acid conjugate	-0.970	0.985	-0.746	0.731	0.955	0.858	1.690	2.297	2.337	3.572	0.0172
N-Acetyl-L-alanine (NAc-L-Ala)	Amino acid conjugate	-0.669	0.481	-0.979	1.167	0.648	0.197	0.360	0.661	-0.844	0.529	0.6121
DLH	Peptide	-0.943	1.311	-0.587	0.218	-1.504	-1.590	-1.395	-1.261	-1.927	-1.639	0.0055
DLS	Peptide	-1.126	0.828	-0.558	0.857	0.017	-0.147	-0.373	-0.032	-0.136	0.469	0.9380
HVDD	Peptide	-0.841	1.071	-0.863	0.633	-0.347	-0.426	-1.217	-0.961	-1.155	-0.702	0.1046
LD	Peptide	-0.067	1.388	-0.974	-0.347	0.886	0.887	0.574	0.718	-0.236	0.552	0.2448
PD	Peptide	-0.754	0.544	-0.923	1.133	-0.798	-0.522	-0.631	-0.220	-0.188	1.584	0.8335
PE	Peptide	-0.649	0.860	-1.059	0.848	0.704	-0.367	0.387	0.525	-0.710	1.134	0.6127
PEE	Peptide	-0.779	1.068	-0.923	0.634	0.392	0.253	0.512	1.102	-0.543	0.563	0.4524
PEEK	Peptide	-0.815	0.507	-0.854	1.161	2.276	1.975	1.258	1.905	1.033	4.231	0.0169
PVNF	Peptide	-0.923	1.294	-0.635	0.264	-0.147	-0.120	-0.089	-0.032	-1.038	-0.784	0.4389
SD	Peptide	-0.685	1.101	-0.993	0.577	-0.045	-0.076	0.162	0.544	-0.141	0.839	0.6428
VD	Peptide	-0.463	1.069	-1.157	0.551	0.869	0.350	0.571	0.171	-0.221	1.496	0.3111
Biotin	Vitamin/cofactor	-0.708	1.402	0.034	-0.729	1.458	2.460	1.325	1.461	1.196	0.897	0.0156
Folate	Vitamin/cofactor	-0.423	-0.773	1.466	-0.270	2.430	2.492	4.422	5.361	5.072	3.248	0.0011
Pantothenate	Vitamin/cofactor	-0.721	1.475	-0.480	-0.274	1.482	1.254	0.526	0.939	0.323	0.197	0.1372

Metabolite compound classes and individual metabolite *z*-scores (see **Materials and Methods** for z-score derivation). P values were determined using one-way ANOVA. Corresponding metabolite signal intensities are reported in S5 Table. Metabolites were harvested on three independent occasions, yielding 4 and 6 individual replicates (r) for the $GC03^{Dd2}$ and $GC03^{Cam734}$ strains, respectively. P values <0.05 are shaded in gray.
