# Supplementary material for: Evolution of Fitness Cost-Neutral Mutant PfCRT Conferring P. falciparum 4-Aminoquinoline Drug Resistance Is Accompanied by Altered Parasite Metabolism and Digestive Vacuole Physiology
Source: PLoS Pathog. 2016 Nov 10;12(11):e1005976. doi: 10.1371/journal.ppat.1005976 (PMC5104409; doi:10.1371/journal.ppat.1005976)
Supplement: S7 Table — For the indicated isogenic, pfcrt-modified lines, digestive vacuole (DV) volume size and pH were determined using spinning disk confocal microscopy and single-cell photometry, respectively, as detailed in Supplementary Materials and Methods. Measurements were made following 30 min exposure to no drug or 2× CQ LD50. Results are reported as mean ± SEM DV volume size (μm3) or pH, as determined for ≥20 parasites, beginning in each case with tightly synchronized young trophozoites. DV volume values for CQ-treated parasites were compared against those of untreated controls to determine the percent increase in size. (PDF) [file ppat.1005976.s014.pdf]

S7 Table. Digestive vacuole volume size and pH of *pfcrt*-modified lines.

	DV volume size ± SEM (μm^3)			pH ± SEM	
**Line**	**No drug**	**CQ**	**% increase**	**No drug**	**CQ**
GC03^{GC03}	1.92 ± 0.04	2.16 ± 0.72	13%	5.74 ± 0.01	5.70 ± 0.01
GC03^{Cam734}	2.52 ± 0.03	3.10 ± 0.17	23%	5.55 ± 0.02	5.54 ± 0.01
GC03$^{Cam734\ F144A}$	2.34 ± 0.02	2.75 ± 0.21	17%	5.61 ± 0.02	5.61 ± 0.01
GC03^{Dd2}	2.79 ± 0.05	3.72 ± 0.13	33%	5.24 ± 0.01	5.28 ± 0.02

For the indicated isogenic, *pfcrt*-modified lines, digestive vacuole (DV) volume size and pH were determined using spinning disk confocal microscopy and single-cell photometry, respectively, as detailed in **Supplementary Materials and Methods**. Measurements were made following 30 min exposure to no drug or 2× CQ LD_{50}. Results are reported as mean ± SEM DV volume size (μm^3) or pH, as determined for ≥20 parasites, beginning in each case with tightly synchronized young trophozoites. DV volume values for CQ-treated parasites were compared against those of untreated controls to determine the percent increase in size.
